# Polyphenol-Rich Fruit Beverage Extracts Reduce Cytokine Secretion in THP-1 Cells

**DOI:** 10.3390/nu18101633

**Published:** 2026-05-21

**Authors:** Lea S. Edrich, Mats Kiene, Leona Heinke, Christian Tesch, Simone Stegmüller, Peter Winterhalter, Elke Richling

**Affiliations:** 1 Division of Food Chemistry and Toxicology, Department of Chemistry, RPTU University Kaiserslautern-Landau, Erwin-Schrödinger-Straße 52, D-67663 Kaiserslautern, Germany; lea.edrich@chem.rptu.de (L.S.E.); simone.stegmueller@chem.rptu.de (S.S.); 2 Institute of Food Chemistry, Technische Universität Braunschweig, Schleinitzstraße 20, D-38106 Braunschweig, Germany; m.kiene@tu-braunschweig.de (M.K.); l.heinke@tu-braunschweig.de (L.H.); c.tesch@tu-braunschweig.de (C.T.); p.winterhalter@tu-braunschweig.de (P.W.)

**Keywords:** red fruits, cytokines, anti-inflammatory, polyphenols, anthocyanins, Amberlite^®^ XAD-7 adsorber resin, Folin–Ciocalteu, HPLC-DAD-ESI-MS^n^, Lumit^®^ Immunoassay

## Abstract

**Background/Objectives.** Inflammation, comprising many complex and finely coordinated immunological processes, represents a vital protective mechanism of the human body. By regulating inflammatory processes, cytokines play a key role in the modulation of the immune system. Secondary plant compounds such as polyphenols influence cellular immunological processes which might contribute to ensuring a physiologically healthy immune status. **Methods/Results.** This study investigated eleven polyphenol-rich extracts from red fruit beverages in terms of potential inhibitory effects on pro-inflammatory cytokine secretion of leukemic monocyte THP-1 cells. Extracts originating from fruit juice (apple), fruit juice concentrate (red grape, black currant, pomegranate, elderberry, aronia), fruit juice puree (cranberry, blueberry) or fruit juice pulp (strawberry, sour cherry) were obtained by adsorption onto Amberlite^®^ XAD-7 resin. The Folin–Ciocalteu assay showed a high content of phenolic compounds in the eleven extracts and HPLC-DAD-ESI-MS^n^ analysis revealed that the extracts contained various anthocyanins in addition to copigments and polymers. Further screening using Lumit^®^ Immunoassay showed that all tested extracts caused a reduction in pro-inflammatory cytokine secretion (interleukins (IL): IL-1β, IL-6, IL-8 and tumor necrosis factor TNF-α). The extracts from red grape and black currant were the most active ones. **Conclusions.** Overall, our results showed that polyphenol-rich fruit extracts can inhibit inflammatory processes in vitro. In vivo studies on the anti-inflammatory effect of fruit juice will be a promising approach to determine the fruit juice-dependent, health-promoting effects in humans.

## 1. Introduction

Inflammation represents a complex immunological defense mechanism characterized by precisely orchestrated cellular interactions that maintain physiologically relevant cellular processes. Immune cells, particularly monocytes and macrophages belonging to the cellular part of the innate immune system, participate in pathogen recognition and elimination, including bacteria and viruses [1]. A variety of immune mediators such as cytokines and chemokines promote inflammatory responses [2,3]. Cytokines can be further subdivided into pro-inflammatory cytokines (e.g., tumor necrosis factor TNF-α and the interleukins (IL) IL-1β, IL-6, IL-8) distinct from their anti-inflammatory counterparts, as for example IL-4 and IL-10 [4,5]. Upon inflammation, pro-inflammatory cytokines play a crucial role in promoting inflammatory processes: TNF-α mainly targets TNF receptors (TNFRs) of macrophages to enhance phagocytosis, IL-8 targets neutrophils via their chemokine receptor (CXCR) to augment chemotaxis, IL-6 targeting IL-6 receptor (IL-6R) reinforces IgG production by B-cell activation and differentiation into plasma cells, while IL-1 activates T-cells, B-cells, and NK-cells to induce their proliferation and differentiation in an IL-1 receptor (IL-1R)-mediated manner [6]. An imbalance in the physiological secretion of inflammation-promoting and -suppressing mediators resulting in an excess of pro-inflammatory cytokine secretion can initiate and enhance the progression of severe life-threatening conditions associated with chronic inflammatory processes [7]. The initiation of pro-inflammatory cytokine secretion can be attributed to intracellular signaling, often including Nuclear Factor kappa-light-chain-enhancer of activated B cells (NF-κB) activation, which represents a key step towards pro-inflammatory propagation [8]. Although NF-κB activation and the following pro-inflammatory cytokine production contribute to a crucial physiological defense mechanism protecting the human body from harmful pathogenic agents, exceeding pro-inflammatory signaling is associated with various inflammatory diseases. Those include cardiovascular diseases, cancer, type 2 diabetes, arthritis, neurodegenerative disorders, and inflammatory bowel disorders such as Crohn’s disease and ulcerative colitis which are known to be related to inflammatory processes [5,9,10]. Chronic inflammation is also associated with increased health complications that have emerged in recent years with COVID-19 [11]. Despite the fact that most of the above-mentioned conditions can be associated with hereditary predispositions, a healthy diet rich in polyphenols plays an important role in prevention [12]. Although the mechanism of action is still elusive, polyphenols may counteract the onset and progression of chronic inflammation [12,13,14]. Beyond their antioxidant, antimutagenic, and chemo-preventive properties, polyphenols, including anthocyanins and copigments (polyphenols without anthocyanins), demonstrate protective effects against inflammatory processes in both in vitro and in vivo studies [10,12,15]. There is evidence that polyphenols modulate immune responses, as it has been shown that apple extract can inhibit the transcription of pro-inflammatory genes in lipopolysaccharide (LPS)-interferon (IFN)-γ-stimulated MonoMac6 cells [16]. A study by Triebel et al. observed that blueberry extract counteracts the translation and secretion of pro-inflammatory cytokines and chemokines in T84 colon cells after IL-1β, IFN-γ, TNF-α stimulation [17]. Another study demonstrated that pomegranate peel extract and its main components, punicalagins and ellagic acid, reduced protein expression of TNF-α, IL-1β, and IL-6 in LPS-stimulated RAW264.7 macrophages [18]. In LPS-stimulated Caco-2 cells, incubation with grape seed extract resulted in the induction of anti-inflammatory gene expression and a decrease in pro-inflammatory cytokines [19]. Hosseini et al. reported significant decreases in IL-6 and C-reactive protein (hs-CRP) indicating anti-inflammatory effects of polyphenols in a human intervention study [20]. In human peripheral blood mononuclear cells (PBMCs) as well as in THP-1 cells, the anthocyanin malvidin was observed to prevent LPS-induced oxidative stress and inflammation [21,22].

This study focused on extracts prepared from eleven polyphenol-rich fruit beverages. The extracts were tested in an in vitro system using the leukemic monocyte cell line THP-1 [23] and the Lumit^®^ Immunoassay. The cells were pretreated with the respective extracts, and the secretion of pro-inflammatory cytokines was monitored. Additionally, the polyphenol compositions of the extracts were determined using HPLC-DAD-ESI-MS^n^.

## 2. Materials and Methods

### 2.1. Chemicals

Double deionized water was prepared using Nanopure^®^ resin (Werner, Leverkusen, Germany). Acetonitrile (LC-MS grade) was obtained from Honeywell Specialty (Seelze, Germany), and formic acid (LC-MS grade) was supplied by Fisher Scientific (Loughborough, UK). Amberlite^®^ XAD-7 HP and 2,2′-azino-bis-(3-ethylbenzothiazoline-6-sulfonic acid) (ABTS, 98%) were obtained from Sigma-Aldrich (Steinheim, Germany). Potassium peroxodisulfate (K_2_S_2_O_8_) was purchased from Riedel-de-Haën (Seelze, Germany). Folin–Ciocalteu’s phenol reagent was obtained from Merck (Darmstadt, Germany). Gallic acid monohydrate (≥98%) and 6-hydroxy-2,5,7,8-tetramethylchroman-2-carboxylic acid (Trolox, ≥98% purity) were obtained from Fluka (Buchs, Switzerland).

### 2.2. Samples

The following samples were used for XAD-7 extract preparation. Aronia (*Aronia melanocarpa*) and the two used red grape (RG) juice concentrates (both *Vitis vinifera* L., one grown in Italy and the other originating from Spain) were obtained from Eckes-Granini Group (Nieder-Olm, Germany). Elderberry (*Sambucus nigra*), black currant (*Ribes nigrum*), and pomegranate (*Punica granatum*) juice concentrate as well as strawberry (*Fragaria ananassa*) fruit pulp were purchased from Döhler GmbH (Darmstadt, Germany). Blueberry (*Vaccinium myrtillus* L.) and cranberry (*Vaccinium macrocarpon*) fruit purees were obtained from Niederrhein-Gold Tersteegen GmbH & Co. KG (Moers, Germany). Sour cherry (*Prunus cerasus*) fruit pulp was purchased from Hans Zipperle AG (Merano, Italy), and cloudy apple juice (*Malus domestica*) from Valensina GmbH (Mönchengladbach, Germany).

### 2.3. Cell Lines, Media, and Stimuli

The cell line THP-1 (ATCC TIB-202^™^, USA) was used, and the cell’s growth medium consisted of RPMI 1640 medium (Invitrogen, Germany) supplemented with 10% heat-inactivated (56 °C, 30 min) fetal calf serum (FCS; Invitrogen, Germany) and 1% Penicillin/Streptomycin (Pen-Strep; Invitrogen, Germany). A serum-reduced medium, used to deprive THP-1 cells of cell serum availability in order to further detect TNF-α, IL-1β,IL-6, and IL-8, was composed of RPMI 1640 medium without phenol red and without glutamine (VWR, Belgium) supplemented with 2 mM L-glutamine (Gibco, Germany), 5% heat-inactivated (56 °C, 30 min) FCS (Invitrogen, Germany), and 1% pen-strep (Invitrogen, Germany). Solvents and chemicals used were of analytical grade or met cell culture standards. Lipopolysaccharide (LPS; Sigma Aldrich, Germany) (1 µg/mL) and interferon-γ (IFN-γ; Abcam, UK) (10 ng/mL) were used for stimulation.

### 2.4. Cell Culture

THP-1 cells were stored at 37 °C, 5% CO_2,_ and 95% humidity and grown at a density of 1 × 10^6^ cells/mL in Corning^®^ T-75 flasks (Greiner, Germany). The cell suspension was passaged every 3–5 days by centrifuging (125× *g*-force (g) for 10 min (min) at room temperature (RT)). Subsequently, the pellet was resuspended in fresh growth medium (composed as described in 2.3 Cell Lines, Media, and Stimuli).

### 2.5. Preparation of Phenolic-Rich Fruit Extracts Using Amberlite XAD-7 Adsorber Resin

Fruit extracts were produced using commercially available juices, concentrates, purees, or pulp of the fruits aronia, elderberry, black currant, pomegranate, blueberry, red grape, sour cherry, cranberry, strawberry, and apple. The extracts were prepared according to Niesen et al. [24]. Approximately 1 L of fruit concentrates were applied onto an Amberlite XAD-7 resin column (glass column 100 × 6 cm, resin volume: 2 L), which had been conditioned with 2 L of methanol and then with 2 L of water. To remove carbohydrates, organic acids, and minerals, the column was washed with 4 L double deionized water. The retained phenolic compounds were eluted with 2 L methanol/acetic acid (19:1; *v*/*v*). The loading, washing and elution flow rate was approximately 25 mL/min. Finally, the solvents were evaporated at 40 °C under reduced pressure, and the polyphenol-rich fruit extracts were freeze-dried and weighted.

### 2.6. Folin–Ciocalteu Assay for Determination of the Total Phenolic Content (TPC Assay)

The Folin–Ciocalteu method according to Singleton and Rossi (1965) was used with slight modifications to determine the total phenolic content (TPC) [25]. Results were expressed as gallic acid equivalents (GAE). Fruit beverage extract samples were prepared in double deionized water at a final concentration of 0.1 mg/mL. The gallic acid calibration standards were freshly prepared daily from the stock solution (aqueous solution, 0.5 mg/mL). For each measurement, 200 μL aliquots of the fruit extract, the standard, and a blank sample were mixed with 1 mL Folin–Ciocalteu reagent (diluted 1:10 *v*/*v* from stock solution with water) in a semi-microcuvette. After a further incubation of five minutes, 800 μL of a 7.5% sodium carbonate solution in water was added to each mixture, which was then incubated for 2 h at room temperature. The extinction was measured at *λ* = 760 nm using a spectrophotometer (Jasco, Groß-Umstadt, Germany). The TPC was calculated using the gallic acid calibration curve (11–66 mg/L). Each sample was measured in triplicate, and the results were expressed as grams of gallic acid equivalents per 100 g of dry weight of extract (g GAE/100 g extract).

### 2.7. HPLC-DAD-ESI-MS^n^ Analysis

The HPLC-DAD-ESI-MS^n^ system consisted of a binary HPLC pump (1100 series) and an autosampler (1200 series), both from Agilent Technologies (Waldbronn, Germany) and was equipped with an LC-ESI-MS^n^ ion-trap system (HCT Ultra ETD II, Bruker Daltonics, Bremen, Germany). Mass spectra were acquired in alternating mode with the capillary voltage set at ±3000 V and the capillary exit at ±121.0 V. Drying gas was nitrogen at a temperature of 365 °C, and a 10.0 L/min flow rate with nebulizer pressure of 50 psi and a scan range from *m*/*z* 100 to 2500 in Ultra Scan mode. Compass Hystar Software (version V. 3.2, Bruker Daltonics) was used for analysis and data collection. HPLC separations were performed on a C18 column (Luna 3u, 100 Å, 3 μm, 150 mm × 2.0 mm i.d.) from Phenomenex (Aschaffenburg, Germany) with a guard column of the same material at a flow rate of 0.20 mL/min. The mobile phase consisted of water/acetonitrile/formic acid (96/3/1; *v*/*v*/*v*) (A) and water/acetonitrile/formic acid (48/51/1; *v*/*v*/*v*) (B). The HPLC conditions for analysis were conducted according to Ostberg-Potthoff et al. [26]: 0 min (6% B), 30 min (35% B), 35 min (40% B), 45 min (90% B), 50 min (90% B), 55 min (30% B), 70 min (6% B), and 80 min (6% B).

### 2.8. THP-1 Stimulation Protocol and Lumit^®^ Immunoassay

THP-1 cells were transferred from their growth medium to a serum-reduced medium (media were composed as described in 2.3 Cell Lines, Media, and Stimuli). A total of 50,000 THP-1 cells were seeded per well in a 96-well suspension plate (Sarstedt, Germany) in order to further detect IL-6 and IL-8, while 200,000 THP-1 cells were seeded per well in a 96-well suspension plate (Sarstedt, Germany) for IL-1β and TNF-α detection. Five hours after seeding, the cells were incubated (37 °C, 5% CO_2_, 95% humidity) for one hour with 50 µg/mL or 100 µg/mL dimethyl sulfoxide (DMSO)-dissolved (final DMSO concentration per well: 0.1% DMSO) XAD-7 fruit extracts (performed for each extract listed in 2.2. Samples). Only 0.1% of DMSO without extract was used as a negative control (no substances reducing inflammatory processes were added). Subsequently, the cells were co-stimulated overnight (18 h, (37 °C, 5% CO_2_, 95% humidity) with 1 µg/mL lipopolysaccharide (LPS) and 10 ng/mL interferon-γ (IFN-γ). The supernatant was transferred into the wells of 96 half-area well plates (Greiner, Germany). The pro-inflammatory cytokine secretion was measured by the photometric (Infinite 500, Tecan, Switzerland, USA) detection of IL-1β, IL-6 and IL-8, TNF-α in the cell culture supernatant using the respective luminescence-based Lumit^®^ (Human) Immunoassay kit from Promega (Promega Corporation, 2800 Woods Hollow Road, Madison, WI 53711-5399, USA) according to the protocol of the manufacturer:

- IL-1β: Technical Manual Lumit^®^ Human IL-1β Immunoassay, Instructions for Use of Products W6010, W6011 and W6012, TM645, Revised 5/25
- IL-6: Technical Manual Lumit^®^ IL-6 (Human) Immunoassay, Instructions for Use of Products W6030, W6031 and W6032, TM685, Revised 5/25
- IL-8: User Guide Lumit^®^ IL-8 (Human) Immunoassay, Instructions for Use of Early Access Material of Product CS2032C02
- TNF-α: Technical Manual Lumit^®^ TNF-α (Human) Immunoassay, Instructions for Use of Products W6050, W6051 and W6052, TM689, Revised 6/25,

All technical literature is available at: https://www.promega.com/protocols/ (accessed on 20 January 2026).

### 2.9. Statistical Analysis

The results of the Lumit^®^ Immunoassay are presented as mean ± standard deviation (SD) of three independent experiments (*n* = 3). One-way ANOVA followed by Tukey’s HSD test was used to assess the overall significance using Excel 2019. Significance relative to the 0.1% DMSO-treated control group was evaluated using a two-sided Student’s *t*-test using Excel 2019, with the significance level set at * *p* < 0.05.

## 3. Results

This study investigated the anti-inflammatory effect of eleven polyphenol-rich fruit beverage extracts, which are an excellent source of polyphenols: aronia (*Aronia melanocarpa*), elderberry (*Sambucus nigra*), black currant (*Ribes nigrum*), pomegranate (*Punica granatum*), blueberry (*Vaccinium myrtillus* L.), red grape (*Vitis vinifera* L., one from Italy and one from Spain), sour cherry (*Prunus cerasus*), cranberry (*Vaccinium macrocarpon*), strawberry (*Fragaria ananassa*), and apple (*Malus domestica*). The extracts were produced using Amberlite^®^ XAD-7 adsorber resin. Using HPLC-DAD-MS^n^ the phenolic composition was evaluated, and the inhibitory potential on pro-inflammatory cytokine secretion in THP-1 cells using the Lumit^®^ Immunoassay kit.

### 3.1. Evaluation of the Phenolic Composition of the Fruit Extracts

**Figure 1:**
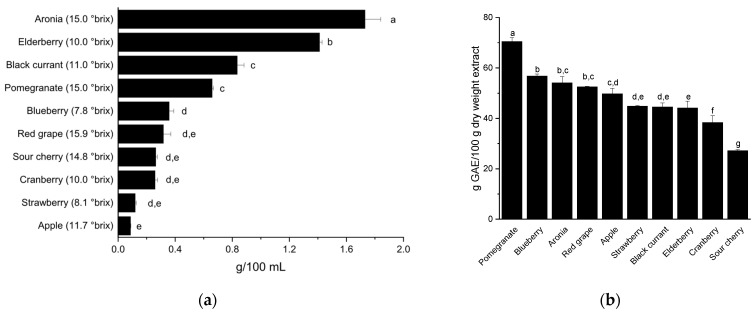
Characterization of the examined fruit extracts: XAD-7 extract yield based on the typical drinking strength (g/100 mL), presented as the mean + SEM with *n* = 2 independent experiments (**a**), and total phenolic content of fruit extracts, determined using the Folin–Ciocalteu assay, presented as the mean + SD with *n* = 3 independent experiments (**b**). The different letters (a–g) indicate significant differences between groups at *p* < 0.05 as measured by Tukey HSD test.

To improve comparability, the XAD-7 extract contents are given at the typical drinking strength of the respective juices (°brix). These are shown in descending order in Figure 1a. The contents ranged from 0.1 to 1.7 g/100 mL. Aronia and elderberry yielded the highest extract amount, whereas the lowest amounts were obtained for strawberry and apple juices. The selected preparation scale made it possible to obtain the required amount of material by producing extracts in duplicate.

The Folin–Ciocalteu assay was used to determine the reducing compounds present in the fruit extracts, including anthocyanins and copigments (polyphenols without anthocyanins). The total phenolic content was calculated in terms of gallic acid equivalents (GAE) and largely corresponds to previously reported studies [27]. All the fruit extracts examined showed high phenolic content, ranging from 27 to 70 g GAE/100 g of extract. The fruit extract of pomegranate had the highest total phenolic content with 70 ± 2 g GAE/100 g extract (Figure 1b). The fruit extracts of blueberry and aronia showed a relatively high total phenolic content of 57 ± 1 and 54 ± 3 g GAE/100 g extract, respectively. The fruit extract of sour cherry revealed the lowest total polyphenol content with 27 ± 1 g GAE/100 g extract. The total phenolic content of red grape from Italy (indicated as red grape in Figure 1) (53 ± 1 g GAE/100 g extract), apple (50 ± 2 g GAE/100 g extract), strawberry (45 ± 1 g GAE/100 g extract), black currant (45 ± 2 g GAE/100 g extract), elderberry (44 ± 3 g GAE/100 g extract), and cranberry (38 ± 3 g GAE/100 g extract) were at similar levels.

The anthocyanin profiles of the fruit extracts, determined by HPLC-DAD-ESI-MS^n^, showed considerable variation (a complete list of compounds identified in the various fruit extracts is included in the Supplementary Materials Figures S1–S11, Tables S1–S21). Pomegranate extract exhibited one of the simplest profiles. It contained cyanidin and delphinidin glucosides (Table 1). In contrast, blueberry and red grape from Italy (indicated as red grape in Figure 1) extracts are characterized by complex anthocyanin profiles. For example, delphinidin, cyanidin, malvidin, peonidin, and petunidin derivatives were identified in those fruit extracts. Blackcurrant extract also contains only a simple but unique anthocyanin profile, consisting mainly of cyanidin and delphinidin rutinosides. The most frequently identified anthocyanins in the investigated fruit extracts were cyanidin-3-glucoside and delphinidin-3-glucoside. Pelargonidin derivatives, on the other hand, are more specific to strawberries. However, no anthocyanins could be detected in the apple fruit extract.

In contrast to anthocyanins, the composition of copigments in those fruit extracts, determined by HPLC-DAD-ESI-MS^n^, varies considerably (Table 2). Pomegranate extract, for example, contains punicalagin, pedunculagin, and ellagic acid. Bilberry, red grape, and blackcurrant extracts, on the other hand, contain myricetin hexosides and quercetin derivatives. Red grapes also contain flavan-3-ols and procyanidins. The composition of apple extract also differs significantly; it contains phloridzin, quercetin derivatives, and chlorogenic acid.

### 3.2. In Vitro Investigations on Polyphenol-Rich Fruit Beverage Extracts on Cytokine Secretion

**Figure 2:**
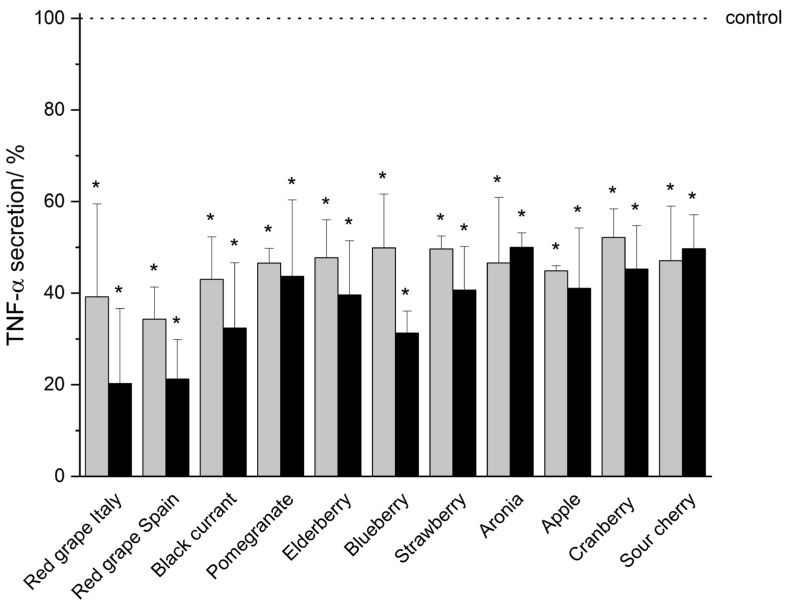
TNF-α secretion after incubation of THP-1 cells with eleven fruit beverage-derived extracts (50 μg/mL: gray bars; 100 μg/mL: black bars) assessed by Lumit^®^ Immunoassay. Data are shown as mean + SD relative to the 0.1% DMSO control (* *p* < 0.05, measured by two-sided Student’s *t*-test), based on *n* = 3 independent experiments.

**Figure 3:**
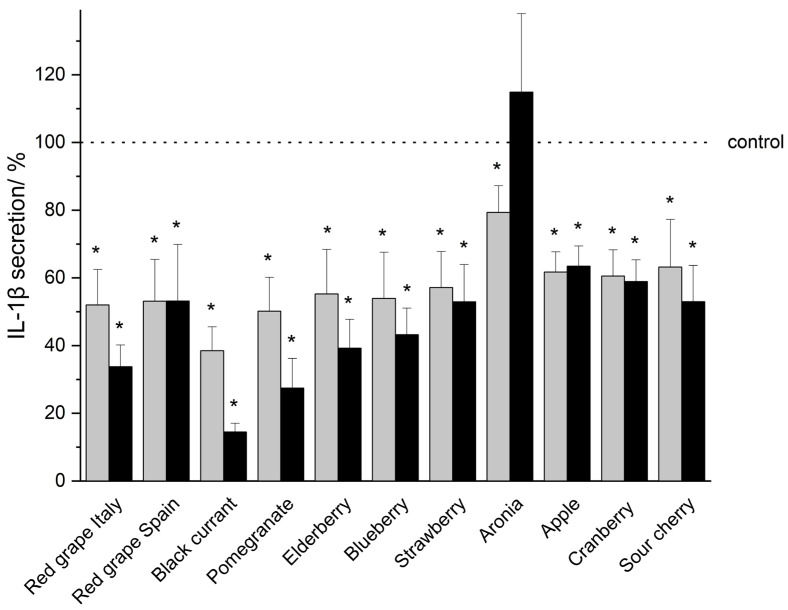
IL-1β secretion after incubation of THP-1 cells with fruit beverage-derived extracts (50 μg/mL: gray bars; 100 μg/mL: black bars) assessed by Lumit^®^ Immunoassay. Data are shown as mean + SD relative to the 0.1% DMSO control (* *p* < 0.05, measured by two-sided Student’s *t*-test), based on *n* = 3 independent experiments.

**Figure 4:**
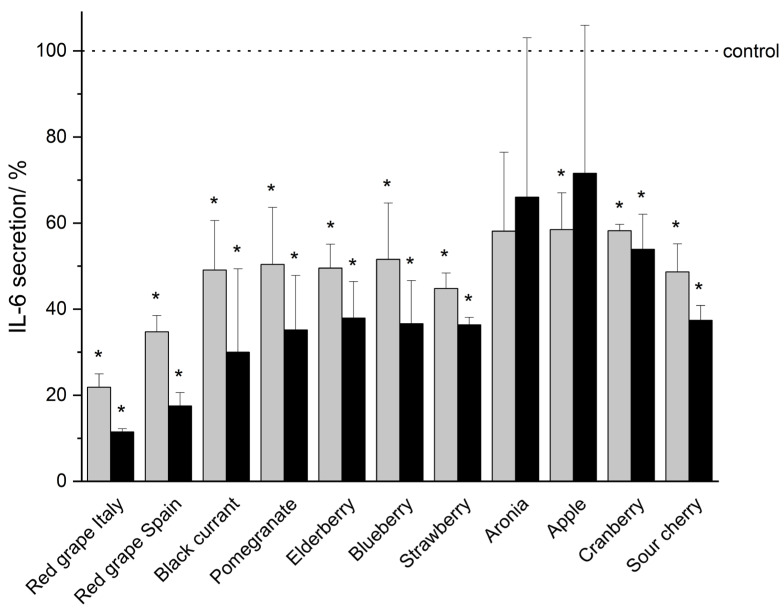
IL-6 secretion after incubation of THP-1 cells with fruit beverage-derived extracts (50 μg/mL: gray bars; 100 μg/mL: black bars) assessed by Lumit^®^ Immunoassay. Data are shown as mean + SD relative to the 0.1% DMSO control (* *p* < 0.05, measured by two-sided Student’s *t*-test), based on *n* = 3 independent experiments.

**Figure 5:**
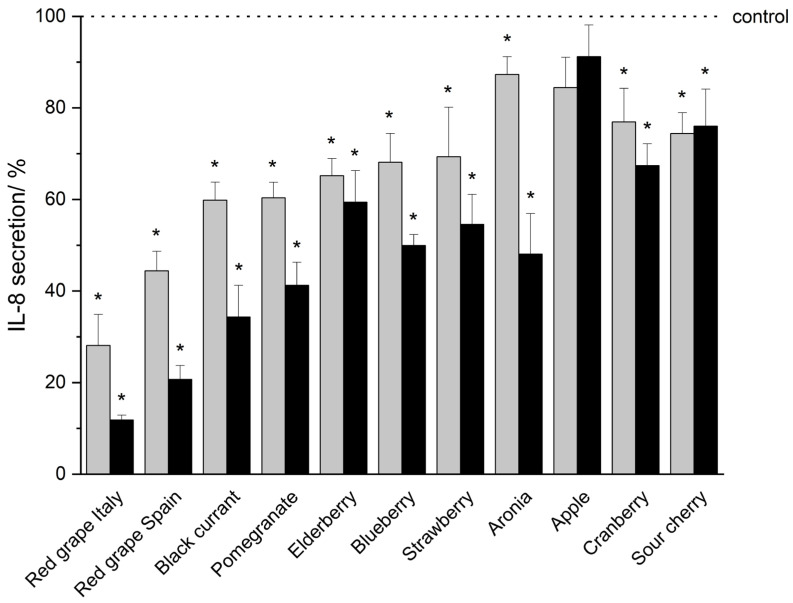
IL-8 secretion after the incubation of THP-1 cells with fruit beverage-derived extracts (50 μg/mL: gray bars; 100 μg/mL: black bars) assessed by Lumit^®^ Immunoassay. Data are shown as mean + SD relative to the 0.1% DMSO control (* *p* < 0.05, measured by two-sided Student’s *t*-test), based on *n* = 3 independent experiments.

In vitro studies demonstrated that THP-1 monocytic cells secrete the pro-inflammatory mediators TNF-α, IL-1β, IL-6, and IL-8 in a serum-reduced environment (5% FCS, 0.1% DMSO) upon LPS-IFN-γ co-stimulation (shown as a dotted line indicated as control in Figure 2, Figure 3, Figure 4 and Figure 5). An incubation of the THP-1 cells with fruit beverage-derived extracts (listed in 2.2 Samples, each one dissolved in 0.1% DMSO) dose-associated (100 μg/mL or 50 μg/mL) inhibited the measured cytokine secretion upon LPS-IFN-γ co-stimulation regarding TNF-α (Figure 2), IL-1β (Figure 3), IL-6 (Figure 4), and IL-8 (Figure 5), each one relative to the corresponding 0.1% DMSO control (Figure 2, Figure 3, Figure 4 and Figure 5). Extract concentrations used are proven to not be cytotoxic on THP-1 cells, which was proven in the resazurin reduction assay (details and data can be found in the Supplementary Materials, Section S2, Figures S12 and S13). Significant marks (* *p* < 0.05) are shown in the figures (Figure 2, Figure 3, Figure 4 and Figure 5) and all *p*-values are shown separately in tabular outline in the Supplementary Materials (Tables S22 and S23). Additionally, two tables consolidating all cytokine inhibition by all extracts tested can be found in the Supplementary Materials (Tables S24 and S25). In the following, “>” is used to distinguish between fruit beverage extracts with higher and lower anti-inflammatory effects (extract leading to a higher percentage decrease in measured cytokine secretion > extract leading to a smaller percentage decrease in measured cytokine secretion).

All eleven tested fruit beverage extracts, each in the concentrations of 50 μg/mL and 100 μg/mL significantly reduced TNF-α secretion (Figure 2, Tables S22–S25). Overall (apart from aronia and sour cherry), an extract concentration of 100 μg/mL led to a stronger inhibition of cytokine secretion than an extract concentration of 50 μg/mL (Figure 2). The TNF-α secretion of treated THP-1 cells was most strongly inhibited by 100 μg/mL red grape extract from Italy (20.27% ± 16.36%, *p* = 0.01375) relative to the 0.1% DMSO control (Figure 2). The second strongest inhibition of TNF-α secretion was reached by 100 μg/mL red grape extract from Spain (21.21% ± 8.64%, *p* = 0.00399, Figure 2). This means that both of the two tested red grape extracts (from Italy and Spain) led to a > 75% decrease in TNF-α secretion relative to the control. A 100 μg/mL blueberry extract reduced TNF-α secretion (*p* = 0.00163) to 31.27% ± 4.81% (>67% decrease), and also a 100 μg/mL black currant extract reduced TNF-α secretion by more than two-thirds (>67% decrease) relative to the control (Figure 2, Tables S22–S25). Furthermore, all other extracts tested in a concentration of 100 μg/mL led to the decrease in cytokine secretion by more than 50% (red grape Italy > red grape Spain > blueberry > black currant > elderberry > strawberry > apple > pomegranate > cranberry > sour cherry > aronia) (see Figure 2, Tables S22–S25).

In the case of IL-1β, 100 μg/mL black currant extract (14.46% ± 2.61%, *p* = 0.00031), 100 μg/mL pomegranate extract (27.45% ± 8.76%, *p* = 0.00482), and 100 μg/mL red grape extract from Italy (33.74% ± 6.42%, *p* = 0.00311, Figure 3, Tables S22–S25) were observed to be the extracts which most strongly inhibit cytokine secretion. Except for the aronia extract, all further tested fruit beverage extracts in the concentration of 100 μg/mL reduced IL-1β secretion significantly by more than one third (>33% decrease) compared to the control (black currant > pomegranate > red grape Italy > elderberry > blueberry > strawberry > sour cherry > red grape Spain > cranberry > apple > aronia) (see Figure 3, Tables S22–S25). Lesser (except in the case of aronia and apple), but also more significant anti-inflammatory effects (>20% decrease in IL-1β secretion) were observable using the extracts in a concentration of 50 μg/mL instead of 100 μg/mL.

Investigations regarding the cytokine IL-6 underscored the aforementioned highly pronounced anti-inflammatory effect of the red grape extract, as 100 μg/mL red grape extract from Italy (11.46% ± 0.76%, *p* = 0.00001) and 100 μg/mL red grape extract from Spain (17.50% ± 3.10%, *p* = 0.00014, Figure 4, Tables S22–S25) significantly reduced IL-6 secretion by more than 80%. In addition, 100 μg/mL of black currant extract diminished IL-6 secretion by more than 70% (red grape Italy > red grape Spain > black currant > pomegranate > strawberry > blueberry > sour cherry > elderberry > cranberry > aronia > apple) (see Figure 4, Tables S22–S25). Except for aronia and apple, the higher extract concentration (100 μg/mL in comparison to 50 μg/mL) came along with a higher percentage of decrease of cytokine secretion (Figure 4, Tables S22–S25).

IL-8 secretion decreased upon treatment with each extract investigated (red grape Italy > red grape Spain > black currant > pomegranate > aronia > blueberry > strawberry > elderberry > cranberry > sour cherry > apple, Figure 5, Tables S22–S25). Again, the higher extract concentration (100 μg/mL in comparison to 50 μg/mL, except for apple and sour cherry) resulted in a more predominant percentage decrease in measured cytokine secretion (Figure 5, Tables S22–S25). Specifically, the red grape from Italy (11.84% ± 1.04%, *p* = 0.00005) and the red grape from Spain (20.67% ± 3.07%, *p* = 0.00050), both at a concentration of 100 μg/mL, followed by 100 μg/mL black currant extract (34.32% ± 6.94%, *p* = 0.00370) efficiently and significantly reduced cytokine secretion (Figure 5, Tables S22–S25).

In general, a reduction in cytokine secretion of THP-1 cells due to polyphenol-rich extract treatment was observed, while the extent of this anti-inflammatory effect varied among the cytokines (TNF-α (Figure 2), IL-1β (Figure 3), IL-6 (Figure 4), and IL-8 (Figure 5), Tables S22–S25). The inhibition of cytokine secretion also depended on the extracts that were tested, as well as on the respective extract concentration used (100 μg/mL or 50 μg/mL, Figure 2, Figure 3, Figure 4 and Figure 5, Tables S22–S25).In summary, the extracts from red grapes (Italy > Spain) and the extract from black currant showed the strongest anti-inflammatory potential of all fruits examined (Figure 2, Figure 3, Figure 4 and Figure 5, Tables S22–S25).

## 4. Discussion

In this study, we examined eleven polyphenol-rich extracts obtained from fruit juice (apple), fruit juice concentrate (red grape Italy, red grape Spain, black currant, pomegranate, elderberry, aronia), fruit juice puree (cranberry, blueberry) or fruit juice pulp (strawberry, sour cherry). As a first step, polyphenol-rich extracts were prepared from fruit juice, concentrates, purees, and pulp using Amberlite^®^ XAD-7 adsorbent resin. The extract yields achieved and shown in Figure 1a were consistent with those reported in the studies by Göttel et al. [31] and Niesen et al. [24].

The Folin–Ciocalteu assay was used to determine the reducing compounds present in the fruit extracts, which are approximately indicated as TPC (Figure 1b). The determination of the TPC showed that the fruit extract of pomegranate had the highest content of total polyphenols at 70 ± 2 g GAE/100 g extract. The extracts from blueberry and aronia also had a relatively high total phenolic content with 57 ± 1 and 54 ± 3 g GAE/100 g extract, respectively. The extract from sour cherry showed the lowest content of total polyphenols with 27 ± 1 g GAE/100 g extract. In an earlier experiment, the TPC content of pomegranate fruit extract was determined by a similar method (67 g GAE/100 g extract) and was in line with the data we achieved [43]. A comparison with two other studies also showed that the determined TPC values are in a similar range. In a study by Berger et al. [27], aronia was found to have the highest value at 65 g GAE/100 g, followed by red grape at 62 g GAE/100 g and pomegranates at 60 g GAE/100 g. Whereas sour cherries had the lowest total polyphenol content at 40 g GAE/100 g. In addition, Köpsel et al. [28] reported that pomegranate, followed by black currant and sour cherry had the highest total polyphenol content. Furthermore, values for the TPC content in fruit juices can also be found in the literature. These values are significantly lower compared to the extracts but they show comparable trends. TPC values of fruit juices varied from 1.2 mg GAE/mL (cranberry juice), over 2 mg GAE/mL (sour cherry juice) and 5 mg GAE/mL (elderberry juice) up to 9 mg GAE/mL (chokeberry and pomegranate juice) [44,45,46,47]. However, when comparing TPC with values from the literature, it is important to take into account that the type and variety of fruit, as well as pre-harvest practices, ripeness, post-harvest storage, and processing methods influence the polyphenol amount of the fruit juices. Therefore, it can be concluded that the production of XAD-7 extracts yielded highly concentrated polyphenol mixtures that can serve as the basis for subsequent activity-guided isolation.

Based on previous studies, it has become increasingly evident that polyphenols are healthy components of our daily nutrition [12,14,21]. Fruit-derived beverages represent substantial sources of phenolic compounds, which reveal various biological activities encompassing antioxidant, antimutagenic, and chemo-preventive functionalities [48]. Dietary patterns characterized by elevated polyphenol consumption exhibit pronounced anti-inflammatory properties, potentially contributing to the attenuation of inflammatory processes [49]. This indicates that our research is contextualized within the growing evidence of anti-inflammatory properties of polyphenols and their potential role in preventing chronic diseases. The role of polyphenols in immunomodulation has been extensively validated through both in vitro and in vivo studies [16,17,18,20,48]. Specifically, a red fruit extract, such as blueberry extract, counteracts the translation and secretion of pro-inflammatory cytokines and chemokines in T84 colon cells [17], which is in line with our findings in THP-1 cells. The anti-inflammatory effect of apple extract [16] and pomegranate extract [18,20] documented in vitro [16,18] and in vivo [20] also aligns with our investigations on THP-1 cells. Extending these observations, our in vitro experiments demonstrate that polyphenol-rich extracts originating from eleven different fruits (red grape from Italy, red grape from Spain, black currant, pomegranate, elderberry, blueberry, strawberry, aronia, apple, cranberry, sour cherry) modulate immunological processes as they inhibit the secretion of the inflammatory key mediators TNF-α, IL-1β, IL-6, and IL-8. This is an essential step towards understanding the molecular mechanisms by which polyphenol-rich foods exert their health-promoting effects. The monocytic THP-1 cell model has proven particularly valuable for studying inflammatory responses, as it allows the investigation on the complex interactions between polyphenols and cytokine secretion under controlled conditions [21,23]. In the context of chronic inflammation, polyphenols are already assumed to prevent inflammation-related diseases, e.g., intestinal inflammatory bowel disease (IBD) which is associated with an excessive release of pro-inflammatory cytokines and chemokines, such as TNF-α and IL-1β [16,50]. We showed that in addition to TNF-α and IL-1β also IL-6 and IL-8 secretion levels of THP-1 cells are significantly reduced due to the polyphenol-rich extract treatment. Because the analyzed cytokines have enormous physiological importance, their downregulation may lead to reduced receptor-mediated immune activation via TNFR, CXCR, IL-6R, or IL-1R, affecting various immune cell types, e.g., macrophages, neutrophils, B-cells, T-cells, and NK-cells, which can alter different immunological processes such as phagocytosis, chemotaxis, IgG production, B-cell differentiation, the activation of T-cells, B-cells and NK-cells [6,50]. This implies the far-reaching impact of polyphenol-induced reduction in cytokine secretion, as well as the need for further research which includes other immune cell types. Additionally, the investigation of intracellular signaling pathways leading to inflammatory gene expression resulting in cytokine secretion seems to be a promising way to further understand polyphenol-dependent anti-inflammation. Although specific mechanisms by which polyphenols modulate the cellular secretion of immune mediators are not elucidated yet, one can hypothesize that cell treatment with the polyphenol-rich extracts interferes with intracellular inflammatory key signaling cascades, such as the NF-κB pathway [8]. As a master transcription factor in both innate and adaptive immunity, NF-κB orchestrates pro-inflammatory gene expression, while deregulated NF-κB activation is a feature of various chronic inflammatory conditions [51,52,53]. This shows the special need for research on polyphenol-associated NF-κB-modulation in the context of managing chronic inflammation and associated diseases.

Furthermore, it was observed that the three extracts with the strongest anti-inflammatory properties differed in their polyphenol profiles. The pomegranate extract is characterized by its high content of copigments such as the ellagitannin punicalin and five anthocyanidins, which could be identified [24,28,34,43]. Extracts from pomegranates and their main components, punicalin and ellagic acid, have been demonstrated to reduce the protein expression of the pro-inflammatory cytokines TNF-α, IL-1β, and IL-6 and to downregulate other inflammatory mediators, including NO and PGE_2_, in LPS-simulated RAW264.7 macrophages [18]. In contrast, the fruit extracts of red grapes and blackcurrants showed the strongest anti-inflammatory effects. Anthocyanidins, proanthocyanidins, and derivatives of myricetin, kaempferol, and quercetin, which characteristically appear in red grape extract have been identified [26,28,31]. Black currant extract is characterized by a high content of delphinidin and cyanidin rutinosides, as well as myricetin, kaempferol, and quercetin derivatives [28,32,38,39]. Research conducting in vitro studies on the protective effects of certain anthocyanidins and anthocyanins on inflammatory processes is reported in the literature. It was demonstrated that pretreatment with the anthocyanidin malvidin led to a significant reduction in the expression of IL-6, TNF-α, IL-1β, and COX-2 mRNA, as well as the respective protein secretion, in peripheral blood mononuclear cells (PBMCs) induced by lipopolysaccharide (LPS) [21]. Furthermore, it was demonstrated that cyanidin-3-glycoside and peonidin-3-glycoside significantly inhibited the expression and secretion of IBD-associated pro-inflammatory mediators (TNF-α, IP-10, I-TAC, sICAM-1, GRO-α) in stimulated human colon epithelial cells (T84) [17]. In addition, anti-inflammatory properties were demonstrated in vitro for a number of copigments. As demonstrated by Nallathambi and coworkers, the incubation of LPS-stimulated Caco-2 cells with proanthocyanidin-rich grape seed extract induces gene expression of anti-inflammatory cytokines and decreases proinflammatory cytokines [19]. Procyanidin B_1_ and B_2_ have also been shown to significantly inhibit proinflammatory gene expression and suppress NF-κB, IP-10, IL-8 promoters, and STAT1-dependent signal transduction [16].

Therefore, the identification of specific polyphenols with anti-inflammatory properties could provide new approaches for the prevention and treatment of chronic inflammatory diseases. Therefore, a targeted analysis of anthocyanins, copigments, and polymers may provide structure-related information about anti-inflammatory agents contained in the polyphenol-rich fruit extracts. Certainly, there are several limitations of our in vitro study, such as the usage of only one cell type (THP-1 monocyte), one stimulation protocol (LPS/IFN-γ-costimulated for 18 h), two concentrations of each XAD-7 extract, and the Folin–Ciocalteu assay, which is acknowledged as an approximation for TPC due to the interference from non-phenolic reducing agents. Also, human pharmacokinetic parameters were not part of our study. Therefore, human intervention studies are of major importance regarding the investigation of anti-inflammatory effects of fruit extract consumption in humans. Overall, our screening investigation contributes substantially to supporting polyphenols’ role in modulating inflammatory responses. Future studies may provide profound implications for both preventive medicine and therapeutic intervention strategies.

## 5. Conclusions

The main objective of this study was to investigate the anti-inflammatory activity of fruit beverage extracts. Eleven polyphenol-rich fruit extracts originating from fruit juice (apple), fruit juice concentrate (red grape from Italy, red grape from Spain, black currant, pomegranate, elderberry, aronia), fruit juice puree (cranberry, blueberry) or fruit juice pulp (strawberry, sour cherry) was prepared and characterized. LPS-IFN-γ-induced secretion of pro-inflammatory cytokines of THP-1 cells were inhibited by the polyphenol-rich XAD-7 extracts under investigation. The most potent extracts with the strongest anti-inflammatory effects were derived from red grapes and black currants. Red grape extracts contained a variety of anthocyanin derivatives and characteristic copigments, including caftaric acid, flavan-3-ols, myricetin, kaempferol, and quercetin derivatives. Blackcurrant extract is characterized by a high content of delphinidin and cyanidin rutinosides, as well as the copigments myricetin, kaempferol, and quercetin derivatives. Our work thus makes an important contribution to the field of nutritional immunomodulation by establishing a link between dietary polyphenolic compounds and inflammatory signals of immune cells. Therefore, red grapes and black currants are the most promising candidates, which caused a reduction in pro-inflammatory cytokine secretion, and they will be investigated in a human intervention study on inflammatory markers. In the future we aim to identify the pathway involved in the anti-inflammatory activity reported here and to identify single compounds or classes of substances responsible for those effects.

## Figures and Tables

**Table 1:** Anthocyanin profiles identified in the monitored fruit beverage derived extracts.

Compound	Mode	Ion *m*/*z*	Fragments *m*/*z*	Apple	Aronia [28,29,30]	Black Currant [28,31,32]	Blueberry [31,33]	Cranberry [28,31]	Elderberry [28,31]	Pomegranate [24,34]	RG * Italy [28,31]	RG * Spain [28,31]	Sour Cherry [28,31]	Strawberry [35]
Cyanidin-3-arabinoside	[M]^+^	419	287		X		X	X						
Cyanidin-3-(6″-acetoyl)-glucoside	[M]^+^	491	287				X							
Cyanidin-3-(6″-coumaroyl)-glucoside	[M]^+^	595	287								X	X		
Cyanidin-3,5-diglucoside	[M]^+^	611	449/287							X				
Cyanidin-3-galactoside	[M]^+^	449	287		X		X	X						
Cyanidin-3-glucoside	[M]^+^	449	287		X	X	X	X	X	X	X	X		X
Cyanidin-3-(2^G^-glucosyl-rutinoside)	[M]^+^	757	611/287										X	
Cyanidin-3-hexoside-(*epi*)-catechin	[M]^+^	737	575/329/287		X									
Cyanidin-3-pentoside-(*epi*)-catechin	[M]^+^	707	575/329/287		X									
Cyanidin-3-rutinoside	[M]^+^	595	449/287			X							X	
Cyanidin-3-sambubioside	[M]^+^	581	287						X					
Cyanidin-3-xyloside	[M]^+^	419	287		X									
Delphinidin-3-arabinoside	[M]^+^	435	303				X							
Delphinidin-3-(6″-acetoyl)-glucoside	[M]^+^	507	303								X			
Delphinidin-3-(6″-coumaroyl)-glucoside	[M]^+^	611	465/303				X				X	X		
Delphinidin-3,5-diglucoside	[M]^+^	627	303							X				
Delphinidin-3-galactoside	[M]^+^	465	303				X							
Delphinidin-3-glucoside	[M]^+^	465	303			X	X			X	X	X		
Delphinidin-3-rutinoside	[M]^+^	611	465/303			X								
Malvidin-3-arabinoside	[M]^+^	463	331				X							
Malvidin-3-(6″-acetoyl)-glucoside	[M]^+^	535	331				X				X	X		
Malvidin-3-(6″-coumaroyl)-glucoside	[M]^+^	639	331								X	X		
Malvidin-3-galactoside	[M]^+^	493	331				X							
Malvidin-3-glucoside	[M]^+^	493	331				X				X	X		
Pelargonidin-3-(6″-acetoyl)-glucoside	[M]^+^	475	271											X
Pelargonidin-3-glucoside	[M]^+^	433	271							X				X
Pelargonidin-3-(6″-malonyl)-glucoside	[M]^+^	519	271											X
Pelargonidin-3-rutinoside	[M]^+^	579	271											X
Peonidin-3-(6″-acetoyl)-glucoside	[M]^+^	505	301				X				X	X		
Peonidin-3-arabinoside	[M]^+^	433	301					X						
Peonidin-3-(6″-coumaroyl)-glucoside	[M]^+^	609	301			X					X	X		
Peonidin-3-galactoside	[M]^+^	463	301				X	X						
Peonidin-3-glucoside	[M]^+^	463	301				X	X			X	X		
Petunidin-3-(6″-acetoyl)-glucoside	[M]^+^	521	317				X				X	X		
Petunidin-3-(6″-coumaroyl)-glucoside	[M]^+^	625	479/317			X					X	X		
Petunidin-3-galactoside	[M]^+^	479	317				X							
Petunidin-3-glucoside	[M]^+^	479	317				X				X	X		

* RG = red grape.

**Table 2:** Major copigments identified in the monitored fruit beverage derived extracts.

Compound	Mode	Ion *m*/*z*	Fragments *m*/*z*	Apple [36,37]	Aronia [28,29,30]	Black Currant [28,31,32,38,39]	Blueberry [31,33]	Cranberry [28,31,40]	Elderberry [28,41]	Pomegranate [24,34]	RG * Italy [28,31]	RG * Spain [28,31]	Sour cherry [28,31]	Strawberry [42]
Caftaric acid	[M−H]^−^	311	179/149								X	X		
Catechin	[M−H]^−^	289	245/205/179	X							X	X		
Chlorogenic acid	[M−H]^−^	353	191/179/161	X	X		X	X	X				X	
Coumaroyl iridoid hexoside	[M−H]^−^	535	371/329					X						
Ellagic acid	[M−H]^−^	301	257/229							X				
Isorhamnetin-3-rutinoside	[M−H]^−^	623	315										X	
Kaempferol-3-glucoside	[M−H]^−^	447	285/255								X	X		
Kaempferol-3-rutinoside	[M−H]^−^	593	285			X			X					
Myricetin-3-hexoside	[M−H]^−^	479	317			X	X	X			X	X		
Phloridzin	[M−H]^−^	435	273/167	X										
Procyanidin B1	[M−H]^−^	577	451/407/289	X							X	X		
Punicalagin	[M−H]^−^	1083	781/601							X				
Punicalin	[M−H]^−^	781	601							X				
Quercetin-3-galactoside	[M−H]^−^	463	301	X	X		X							
Quercetin-3-glucoronide	[M−H]^−^	477	301				X				X	X		X
Quercetin-3-glucoside	[M−H]^−^	463	301	X	X	X	X	X	X		X	X		
Quercetin-3-rhamnoside	[M−H]^−^	447	301	X			X	X						
Quercetin-3-rutinoside	[M−H]^−^	609	301		X	X	X		X		X	X	X	

* RG = red grape.

## Data Availability

The original contributions presented in this study are included in the article/Supplementary Materials. Further inquiries can be directed to the corresponding author.

## Supplementary Materials

The following supporting information can be downloaded at: https://www.mdpi.com/article/10.3390/nu18101633/s1. References [24,28,29,30,31,32,33,34,35,36,37,38,39,40,41,42,54] are cited in the Supplementary Materials.

## Abbreviations

The following abbreviations are used in this manuscript:

CXCR	chemokine receptor
DMSO	dimethyl sulfoxide
FCS	fetal calf serum
g	g-force
GAE	gallic acid equivalents
HHDP	hexahydroxydiphenoyl
HPLC-DAD-ESI-MS	high-performance liquid chromatography with diode array detector and electrospray ionization mass spectrometry
IFN	interferon
IL	interleukin
LPS	lipopolysaccharide
n. s.	not significant
NF-κB	nuclear factor kappa-light-chain-enhancer of activated B cells
PBMC	peripheral blood mononuclear cell
Pen	penicillin
Strep	streptomycin
RG	red grape
RT	room temperature
SD	standard deviation
TNF	tumor necrosis factor
TPC	total phenolic content
