# Supplementary material for: Polyphenol-Rich Fruit Beverage Extracts Reduce Cytokine Secretion in THP-1 Cells
Source: Nutrients. 2026 May 21;18(10):1633. doi: 10.3390/nu18101633 (PMC13209995; doi:10.3390/nu18101633)
Supplement: Supplementary file 1 [file nutrients-18-01633-s001.zip › nutrients-4155572-supplementary.pdf]

nutrients

Supplementary Materials

Polyphenol-Rich Fruit Beverage Extracts Reduce Cytokine Secretion in THP-1 Cells

Lea S. Edrich [1], Mats Kiene [2], Leona Heinke [2], Christian Tesch [2], Simone Stegmüller [1], Peter Winterhalter [2] and Elke Richling [1,*]

[1] Division of Food Chemistry and Toxicology, Department of Chemistry, RPTU University Kaiserslautern-Landau, Erwin-Schrödinger-Straße 52, D-67663 Kaiserslautern, Germany; lea.edrich@chem.rptu.de (L.S.E.); simone.stegmueller@chem.rptu.de (S.S.)

[2] Institute of Food Chemistry, Technische Universität Braunschweig, Schleinitzstraße 20, D-38106 Braunschweig, Germany; m.kiene@tu-braunschweig.de (M.K.); l.heinke@tu-braunschweig.de (L.H.); c.tesch@tu-braunschweig.de (C.T.); p.winterhalter@tu-braunschweig.de (P.W.)

* Correspondence: elke.richling@chem.rptu.de; Tel.: +49-631-205-4061

Section S1: HPLC-DAD-ESI-MS/MS analysis for each fruit juice extract and complete list of the anthocyanins and copigments detected

Apple

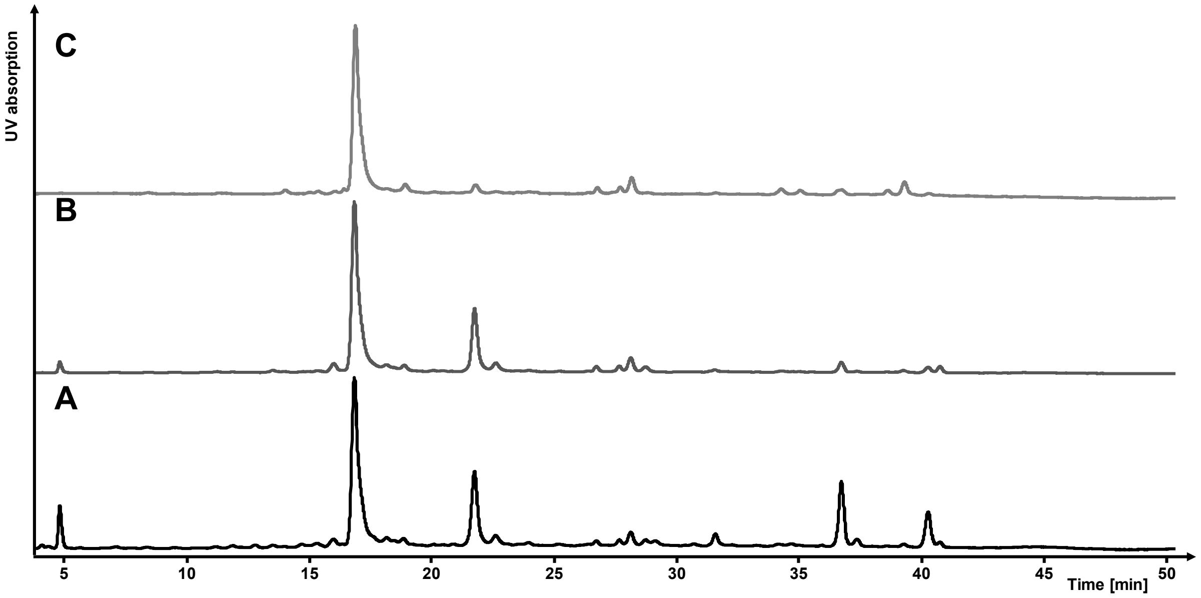

Figure S1. HPLC-UV chromatograms of the XAD-7 extract of apple at a wavelength of λ 280 nm (A), λ 320 nm (B), and λ 360 nm (C). Peak identification is given in Table S1.

Table S1. HPLC-ESI-MS/MS data of the XAD-7 extract of apple, identified copigments and their absorption maxima λ_{max}.

Peak No.	Retention time (min)	$[M–H]^-$ *m*/*z*	Fragments *m*/*z*	λ_{max} (nm)	Copigment	Reference
1	12.9	577	451/425/407/289	280	Procyanidin B_1	[36,37]
2	15.7	289	245/205/179	280	Catechin	[36,37]
3	16.9	353	191/179/161	325	Chlorogenic acid	[36,37]
4	19.1	577	451/425/407/289	280	Procyanidin B_2	[36,37]
5	21.8	289	245/205/179	280	Epicatechin	[36,37]
6	22.0	337	191/173/163	312	4-*p*-Cumaroylquinic acid	[36,37]
7	34.4	463	301	352	Quercetin-3-galactoside	[36,37]
8	35.1	463	301	352	Quercetin-3-glucoside	[36,37]
9	36.8	567	273	284	Phloretin-2′-*O*-xyloglucoside	[36,37]
10	39.4	447	301	347	Quercetin-3-rhamnoside	[36,37]
11	40.4	435	273/167	284	Phloridzin	[36,37]

Aronia

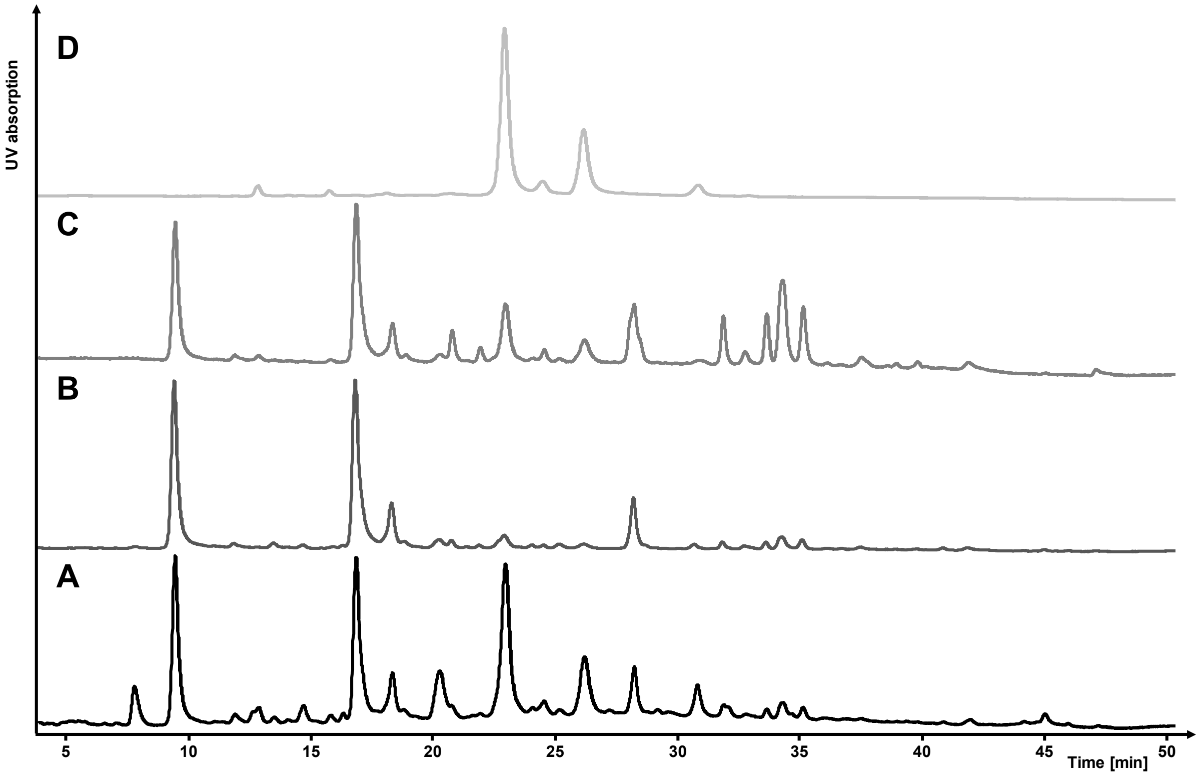

Figure S2. HPLC-UV chromatograms of the XAD-7 extract of aronia at a wavelength of λ 280 nm (A), λ 320 nm (B), λ 360 nm (C), and λ 520 nm (D). Peak identification is given in Tables S2 and S3.

Table S2. HPLC-ESI-MS/MS data of the XAD-7 extract of aronia, identified anthocyanidins and their absorption maxima λ_{max}.

Peak No.	Retention time (min)	$[M]^+$ *m*/*z*	Fragments *m*/*z*	λ_{max} (nm)	Anthocyanin	Reference
A1	13.1	737	575/423/329/287	526/280	Cyanidin-3-hexoside-(*epi*)-catechin	[28–30]
A2	15.9	707	575/423/329/287	526/280	Cyanidin-3-pentoside-(*epi*)-catechin	[29,30]
A3	23.1	449	287	516/280	Cyanidin-3-galactoside	[28–30]
A4	24.7	449	287	516/280	Cyanidin-3-glucoside	[28–30]
A5	26.3	419	287	516/280	Cyanidin-3-arabinoside	[28–30]
A6	31.0	419	287	516/280	Cyanidin-3-xyloside	[28–30]

Table S3. HPLC-ESI-MS/MS data of the XAD-7 extract of aronia, identified copigments and their absorption maxima λ_{max}.

Peak No.	Retention time (min)	$[M–H]^-$ *m*/*z*	Fragments *m*/*z*	λ_{max}	Copigment	Reference
1	9.5	353	191/179/135	324	Neochlorogenic acid	[28,30]
2	17.0	353	191/179/161	325	Chlorogenic acid	[28,30]
3	28.1	625	445/301	352	Quercetin-dihexoside	[28,30]
4	28.6	625	445/301	352	Quercetin-dihexoside	[28,30]
5	31.9	595	301/179/151	347	Quercetin-3-vicianoside	[28,30]
6	33.7	609	301	353	Quercetin-3-robinobioside	[28,30]
7	34.2	609	301	353	Quercetin-3-rutinoside	[28,30]
8	34.5	463	301	352	Quercetin-3-galactoside	[28,30]
9	35.3	463	301	352	Quercetin-3-glucoside	[28,30]

Black currant

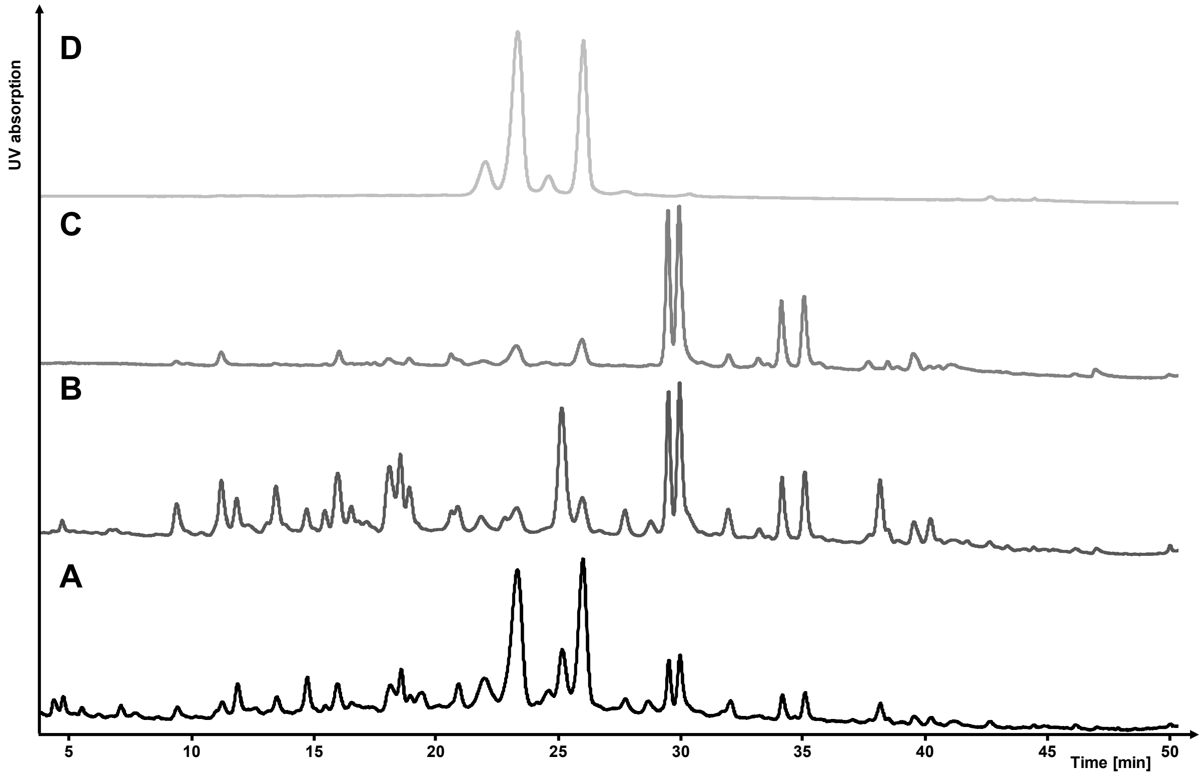

Figure S3. HPLC-UV chromatograms of the XAD-7 extract of black currant at a wavelength of λ 280 nm (A), λ 320 nm (B), λ 360 nm (C), and λ 520 nm (D). Peak identification is given in Tables S4 and S5.

Table S4. HPLC-ESI-MS/MS data of the XAD-7 extract of black currant, identified anthocyanidins and their absorption maxima λ_{max}.

Peak No.	Retention time (min)	$[M]^+$ *m/z*	Fragments *m/z*	λ_{max} (nm)	Anthocyanin	Reference
A1	22.2	465	303	523/280	Delphinidin-3-glucoside	[28,31,32]
A2	23.4	611	465/303	525/280	Delphinidin-3-rutinoside	[28,31,32]
A3	24.8	449	287	516/280	Cyanidin-3-glucoside	[28,31,32]
A4	26.1	595	449/287	517/280	Cyanidin-3-rutinoside	[28,31,32]
A5	28.0	625	479/317	524/280	Petunidin-3-(6′′-coumaroyl)-glucoside	[28,31,32]
A6	30.4	609	301	520/280	Peonidin-3-(6′′-coumaroyl)-glucoside	[32]

Table S5. HPLC-ESI-MS/MS data of the XAD-7 extract of black currant, identified copigments and their absorption maxima λ_{max}.

Peak No.	Retention time (min)	[M–H]⁻ *m/z*	Fragments *m/z*	λ_{max} (nm)	Copigment	Reference
1	9.7	353	191/179/135	324	Neochlorogenic acid	[28,32]
2	11.4	341	179/161	325	Caffeoylhexose	[28,32]
3	13.7	337	163	310	3-*p*-Coumaroylquinic acid	[38]
4	14.9	325	163/145	314	*p*-Coumaroylhexose	[28,32]
	17.3	353	191/179/161	325	Chlorogenic acid	[28,32]
5	19.1	355	193	328	Feruloylhexose	[32]
6	29.7	625	317/316/179	352	Myricetin-3-hexosyl–deoxyhexoside	[32,39]
7	30.1	479	317/316	352	Myricetin-3-hexoside	[32]
8	34.3	609	301	356	Quercetin-3-rutinoside	[28,32]
9	35.2	463	301	352	Quercetin-3-glucoside	[28,32]
10	38.7	593	285	350	Kaempferol-hexosyl–deoxyhexoside	[32,39]

Blueberry

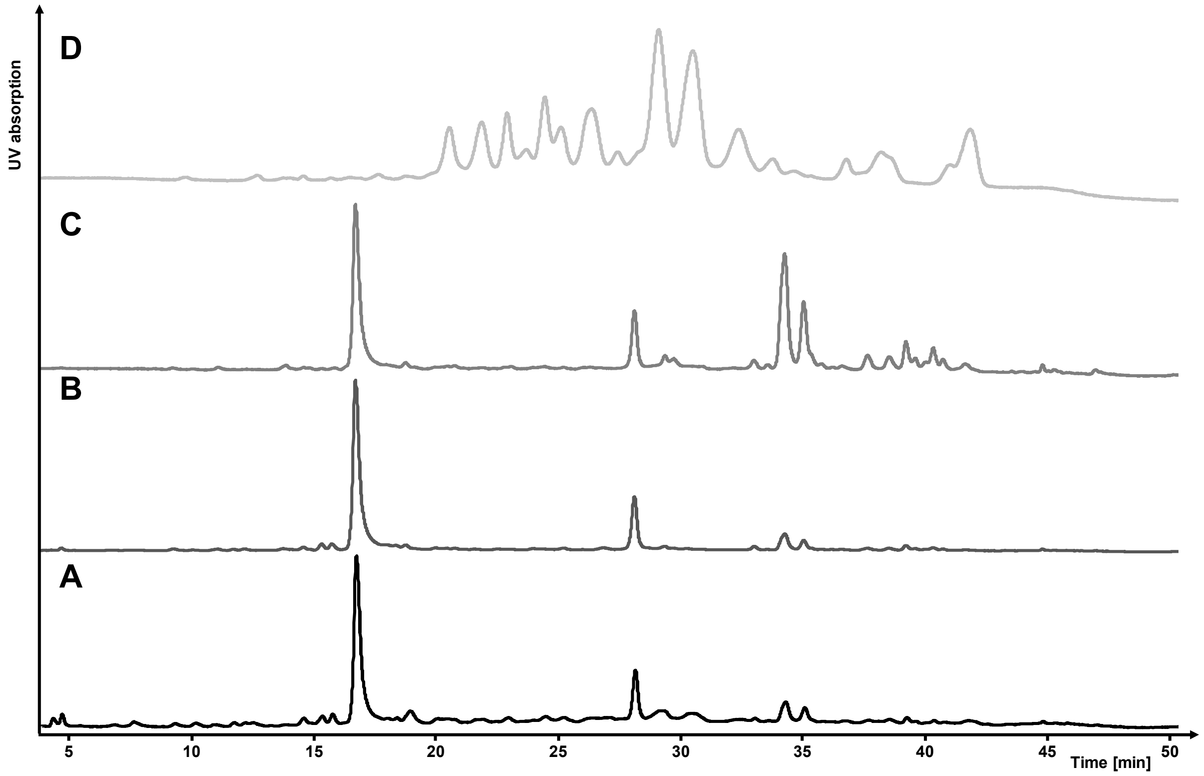

Figure S4. HPLC-UV chromatograms of the XAD-7 extract of blueberry at a wavelength of λ 280 nm (A), λ 320 nm (B), λ 360 nm (C), and λ 520 nm (D). Peak identification is given in Tables S6 and S7.

Table S6. HPLC-ESI-MS/MS data of the XAD-7 extract of blueberry, identified anthocyanidins and their absorption maxima λ_{max}.

Peak No.	Retention time (min)	$[M]^+$ *m/z*	Fragments *m/z*	λ_{max} (nm)	Anthocyanin	Reference
A1	20.7	465	303	523/280	Delphinidin-3-galactoside	[31,33]
A2	22.1	465	303	523/280	Delphinidin-3-glucoside	[31,33]
A3	23.2	449	287	516/280	Cyanidin-3-galactoside	[31,33]
A4	24.0	435	303	523/280	Delphinidin-3-arabinoside	[31,33]
A5	24.7	449	287	516/280	Cyanidin-3-glucoside	[31,33]
A6	25.3	479	317	526/280	Petunidin-3-galactoside	[31,33]
A7	26.3	419	287	516/280	Cyanidin-3-arabinoside	[31,33]
A8	26.7	479	317	526/280	Petunidin-3-glucoside	[31,33]
A9	27.5	463	301	523/280	Peonidin-3-galactoside	[31,33]
A10	29.1	463	301	523/280	Peonidin-3-glucoside	[31,33]
A11	29.4	493	331	526/280	Malvidin-3-galactoside	[31,33]
A12	30.6	493	331	526/280	Malvidin-3-glucoside	[31,33]
A13	32.5	463	331	526/280	Malvidin-3-arabinoside	[31,33]
A14	37.0	491	287	521/280	Cyanidin-3-(6′′-acetoyl)-glucoside	[33]
A15	38.3	521	317	526/280	Petunidin-3-(6′′-acetoyl)-glucoside	[33]
A16	41.0	505	301	523/280	Peonidin-3-(6′′-acetoyl)-glucoside	[33]
A17	41.9	535	331	528/280	Malvidin-3-(6′′-acetoyl)-glucoside	[33]

Table S7. HPLC-ESI-MS/MS data of the XAD-7 extract of blueberry, identified copigments and their absorption maxima λ_{max}.

Peak No.	Retention time (min)	$[M–H]^-$ *m/z*	Fragments *m/z*	λ_{max} (nm)	Copigment	Reference
1	9.5	353	191/179/135	324	Neochlorogenic acid	[31,33]
2	12.7	577	451/425/407/289	280	Procyanidin B_1	[33]
3	15.4	341	179	291	Caffeic acid hexoside	[31,33]
4	15.5	289	245/205/179	280	Catechin	[33]
5	16.7	353	191/179/161	325	Chlorogenic acid	[31,33]
6	18.8	577	451/425/407/289	280	Procyanidin B_2	[33]
7	21.7	289	245/205/179	280	Epicatechin	[33]
8	29.5	479	317/316	352	Myricetin-3-hexoside	[31,33]
9	34.2	609	301	356	Quercetin-3-rutinoside	[31,33]
10	34.5	463	301	352	Quercetin-3-galactoside	[31,33]
11	35.2	463	301	352	Quercetin-3-glucoside	[31,33]
12	35.3	477	301	352	Quercetin-3-glucuronide	[31,33]
13	37.7	433	301	351	Quercetin-pentoside	[31,33]
14	39.4	447	301	347	Quercetin-3-rhamnoside	[31,33]
15	40.8	507	344	343	Syringetin-3-hexoside	[31,33]

Cranberry

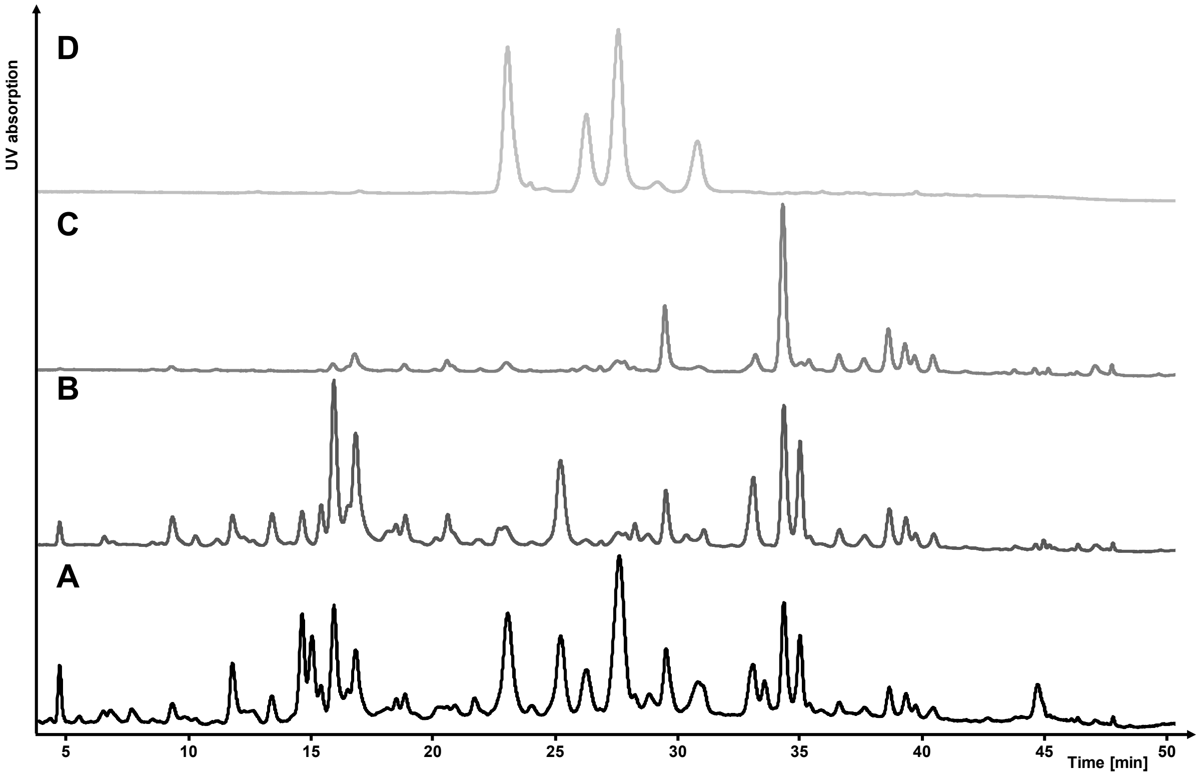

Figure S5. HPLC-UV chromatograms of the XAD-7 extract of cranberry at a wavelength of λ 280 nm (A), λ 320 nm (B), λ 360 nm (C), and λ 520 nm (D). Peak identification is given in Tables S8 and S9.

Table S8. HPLC-ESI-MS/MS data of the XAD-7 extract of cranberry, identified anthocyanidins and their absorption maxima λ_{max}.

Peak No.	Retention time (min)	$[M]^+$ *m/z*	Fragments *m/z*	λ_{max} (nm)	Anthocyanin	Reference
A1	23.2	449	287	516/280	Cyanidin-3-galactoside	[28,31]
A2	24.7	449	287	516/280	Cyanidin-3-glucoside	[28,31]
A3	26.5	419	287	516/280	Cyanidin-3-arabinoside	[28,31]
A4	27.8	463	301	523/280	Peonidin-3-galactoside	[28,31]
A5	29.4	463	301	523/280	Peonidin-3-glucoside	[28,31]
A6	31.0	433	301	521/280	Peonidin-3-arabinoside	[28,31]

Table S9. HPLC-ESI-MS/MS data of the XAD-7 extract of cranberry, identified copigments and their absorption maxima λ_{max}.

Peak No.	Retention time (min)	$[M–H]^-$ *m*/*z*	Fragments *m*/*z*	λ_{max} (nm)	Copigment	Reference
1	9.5	353	191/179/135	324	Neochlorogenic acid	[28]
2	11.4	341	179/161	313	Caffeoylhexose	[28,31]
3	14.8	325	163/145	320	*p*-Coumaroylhexose	[28,31]
4	16.7	325	145	320	*p*-Coumaroylhexose	[31]
5	17.0	353	191/179/161	325	Chlorogenic acid	[28,31]
6	19.1	577	451/425/407/289	280	Procyanidin B_2	[31]
7	20.8	385	223	294	Sinapoylhexose	[31]
8	21.7	289	245/205/179	280	Epicatechin	[40]
9	24.2	865	577/451/407/289	280	Procyanidin trimer B-typ	[31]
10	25.5	337	191	312	Coumaroylquinic acid	[31]
11	29.0	863	711/575/407	280	Procyanidin trimer A-typ	[31]
12	29.7	479	317/316	352	Myricetin-3-hexoside	[28,31]
13	33.2	535	371/329	350	Coumaroyl iridoid hexoside	[28,31]
14	34.8	463	301	352	Quercetin-3-glucoside	[28,31]
15	35.5	493	331	355	Laricitrin-hexoside	[40]
16	36.8	433	301	351	Quercetin-3-pentoside	[28,31]
17	38.7	433	301	351	Quercetin-3-pentoside	[28,31]
18	39.4	447	301	346	Quercetin-3-rhamnoside	[28,31]
19	40.6	507	344	350	Syringetin-hexoside	[28,31]
20	45.3	477	344	354	Syringetin-pentoside	[40]

Elderberry

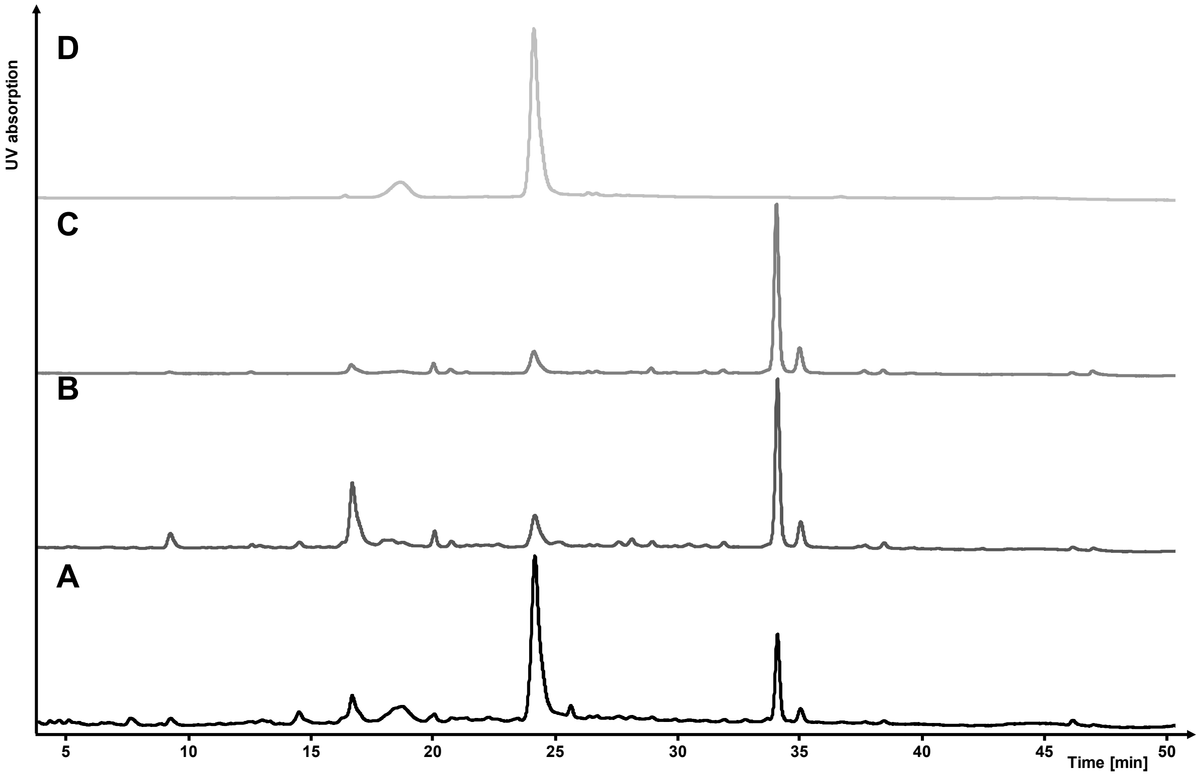

Figure S6. HPLC-UV chromatograms of the XAD-7 extract of elderberry at a wavelength of λ 280 nm (A), λ 320 nm (B), λ 360 nm (C), and λ 520 nm (D). Peak identification is given in Tables S10 and S11.

Table S10. HPLC-ESI-MS/MS data of the XAD-7 extract of elderberry, identified anthocyanidins and their absorption maxima λ_{max}.

Peak No.	Retention time (min)	$[M]^+$ *m/z*	Fragments *m/z*	λ_{max} (nm)	Anthocyanin	Reference
A1	18.3	611	449/287	525/280	Cyanidin-dihexoside	[28,31]
A2	19.1	743	581/449/287	514/280	Cyanidin-3-sambubioside-5-glucoside	[28,31]
A3	24.3	581	287	516/280	Cyanidin-3-sambubioside	[28,31]
A4	24.8	449	287	516/280	Cyanidin-3-glucoside	[28,31]

Table S11. HPLC-ESI-MS/MS data of the XAD-7 extract of elderberry, identified copigments and their absorption maxima λ_{max}.

Peak No.	Retention time (min)	$[M–H]^-$ *m/z*	Fragments *m/z*	λ_{max} (nm)	Copigment	Reference
1	9.4	353	191/179/135	324	Neochlorogenic acid	[28,41]
2	16.8	353	191/179/161	325	Chlorogenic acid	[28,41]
3	17.2	353	191/171	325	Cryptochlorogenic acid	[28,41]
4	34.2	609	301	356	Quercetin-3-rutinoside	[28,41]
5	35.1	463	301	352	Quercetin-3-glucoside	[28,41]
6	28.3	367	191/173	312	5-Feruloylquinic acid	[41]
7	38.6	593	285/255	345	Kaempferol-3-rutinoside	[28,41]
8	39.8	623	315	354	Isorhamnetin-3-rutinoside	[28,41]

Pomegranate

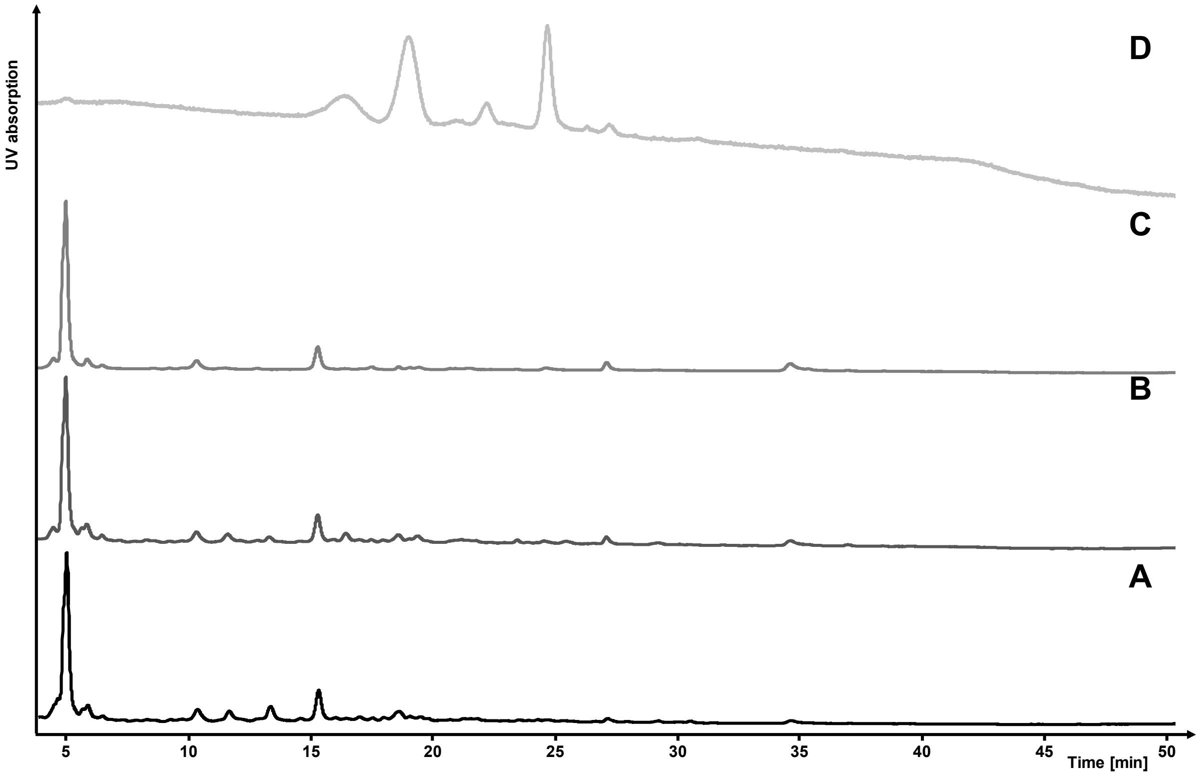

Figure S7. HPLC-UV chromatograms of the XAD-7 extract of pomegranate at a wavelength of λ 280 nm (A), λ 320 nm (B), λ 360 nm (C), and λ 520 nm (D). Peak identification is given in Tables S12 and S13.

Table S12. HPLC-ESI-MS/MS data of the XAD-7 extract of pomegranate, identified anthocyanidins and their absorption maxima λ_{max}.

Peak No.	Retention time (min)	$[M]^+$ *m/z*	Fragments *m/z*	λ_{max} (nm)	Anthocyanin	Reference
A1	16.1	627	303	521/280	Delphinidin-3,5-diglucoside	[24,34]
A2	19.0	611	449/287	525/280	Cyanidin-3,5-diglucoside	[24,34]
A3	20.9	595	433/271	504/280	Pelargonidin-3,5-diglucoside	[34]
A4	22.3	465	303	523/280	Delphinidin-3-glucoside	[24,34]
A5	24.6	449	287	516/280	Cyanidin-3-glucoside	[24,34]
A6	27.0	433	271	504/280	Pelargonidin-3-glucoside	[34]

Table S13. HPLC-ESI-MS/MS data of the XAD-7 extract of pomegranate, identified copigments and their absorption maxima λ_{max}.

Peak No.	Retention time (min)	[M–H]⁻ *m/z*	Fragments *m/z*	λ_{max} (nm)	Copigment	Reference
1	2.9	481	301/275	280	HHDP-hexoside*	[34]
2	3.8	649	605/497/301	376	Trisgalloyl-glucoside	[24,34]
3	4.9	781	721/601/449/299/271	377	Punicalin I	[24,34]
4	5.6	781	721/601/449/299/271	377	Punicalin II	[24,34]
5	9.1	783	721/601/301	377	Pedunculagin I	[24,34]
6	10.3	1083	781/721/601	377	Punicalagin I	[24,34]
7	11.6	951	907/783	377	HHDP-valoneoyl-glucoside*	[24]
8	13.2	783	721/601/301	377	Pedunculagin II	[24,34]
9	15.3	1083	781/721/601	377	Punicalagin II	[24,34]
10	17.0	799	301	374	Granatin	[13]
11	17.6	1085	783/451	375	Digalloyl-gallagyl-hexoside	[24,34]
12	18.4	783	721/601/301	377	Pedunculagin III	[24,34]
13	18.7	801	347	364	Digalloyl-HHDP-glucuronide	[24]
14	21.2	633	301	370	Galloyl-HHDP-glucoside	[24,34]
15	27.0	463	301/257/229	360	Ellagic acid-hexoside	[24,34]
16	34.3	447	300/257	360	Ellagic acid-deoxyhexoside	[34]
17	34.6	301	257/229	366	Ellagic acid	[24,34]

*HHDP = Hexahydroxydiphenoyl

Red grape Italy

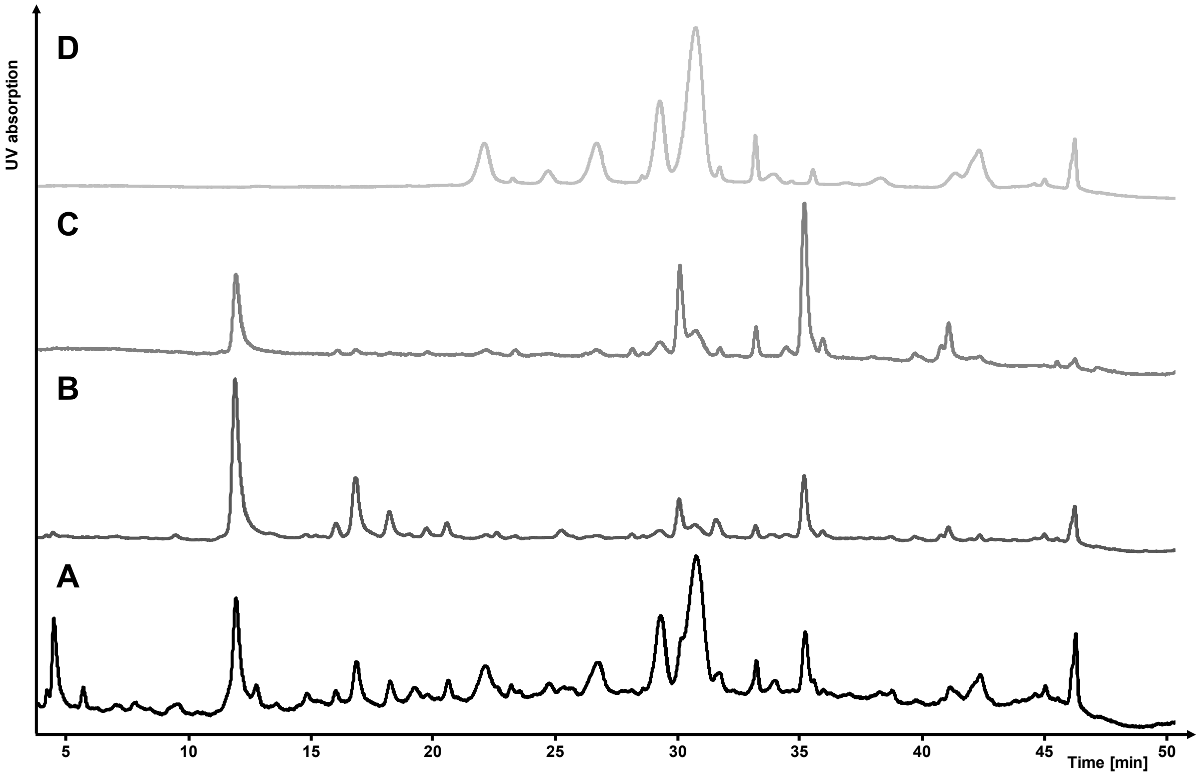

Figure S8. HPLC-UV chromatograms of the XAD-7 extract of red grape (Italy) at a wavelength of λ 280 nm (A), λ 320 nm (B), λ 360 nm (C), and λ 520 nm (D). Peak identification is given in Tables S14 and S15.

Table S14. HPLC-ESI-MS/MS data of the XAD-7 extract of red grape (Italy), identified anthocyanidins and their absorption maxima λ_{max}.

Peak No.	Retention time (min)	$[M]^+$ *m/z*	Fragments *m/z*	λ_{max} (nm)	Anthocyanin	Reference
A1	22.2	465	303	523/280	Delphinidin-3-glucoside	[28,31]
A2	24.9	449	287	516/280	Cyanidin-3-glucoside	[28,31]
A3	26.6	479	317	526/280	Petunidin-3-glucoside	[28,31]
A4	29.4	463	301	523/280	Peonidin-3-glucoside	[28,31]
A5	30.9	493	331	526/280	Malvidin-3-glucoside	[28,31]
A6	34.1	507	303	523/280	Delphinidin-3-(6′′-acetoyl)-glucoside	[31]
A7	36.9	491	287	521/280	Cyanidin-3-(6′′-acetoyl)-glucoside	[31]
A8	38.3	521	317	526/280	Petunidin-3-(6′′-acetoyl)-glucoside	[28,31]
A9	41.2	505	301	523/280	Peonidin-3-(6′′-acetoyl)-glucoside	[28,31]
A10	42.3	535	331	528/280	Malvidin-3-(6′′-acetoyl)-glucoside	[28,31]
A11	42.8	611	303	523/280	Delphinidin-3-(6′′-coumaroyl)-glucoside	[31]
A12	44.6	595	287	521/280	Cyanidin-3-(6′′-coumaroyl)-glucoside	[28,31]
A13	45.0	625	479/317	524/280	Petunidin-3-(6′′-coumaroyl)-glucoside	[28,31]
A14	46.1	609	301	520/280	Peonidin-3-(6′′-coumaroyl)-glucoside	[28,31]
A15	46.2	639	331	530/280	Malvidin-3-(6′′-coumaroyl)-glucoside	[28,31]

Table S15. HPLC-ESI-MS/MS data of the XAD-7 extract of red grape (Italy), identified copigments and their absorption maxima λ_{max}.

Peak No.	Retention time (min)	$[M–H]^-$ *m/z*	Fragments *m/z*	λ_{max} (nm)	Copigment	Reference
1	4.6	169	125	278	Gallic acid	[28,31]
2	12.8	577	451/425/407/289	280	Procyanidin B_1	[28,31]
3	12.0	311	179/149	325	Caftaric acid	[28,31]
4	15.6	289	245/205/179	280	Catechin	[28,31]
5	16.2	325	265/235	314	Coumaric acid hexoside	[28,31]
6	16.9	295	163	312	Coutaric acid	[28,31]
7	19.0	577	451/425/407/289	280	Procyanidin B_2	[28,31]
8	21.9	289	245/205/179	280	Epicatechin	[28,31]
9	30.2	479	317/316	352	Myricetin-3-hexoside	[28,31]
10	34.3	609	301	356	Quercetin-3-rutinoside	[28,31]
11	35.3	463	301	352	Quercetin-3-glucoside	[28,31]
12	35.6	477	301	352	Quercetin-3-glucoronide	[28,31]
13	39.8	447	285/255	345	Kaempferol-3-glucoside	[28,31]

Red grape Spain

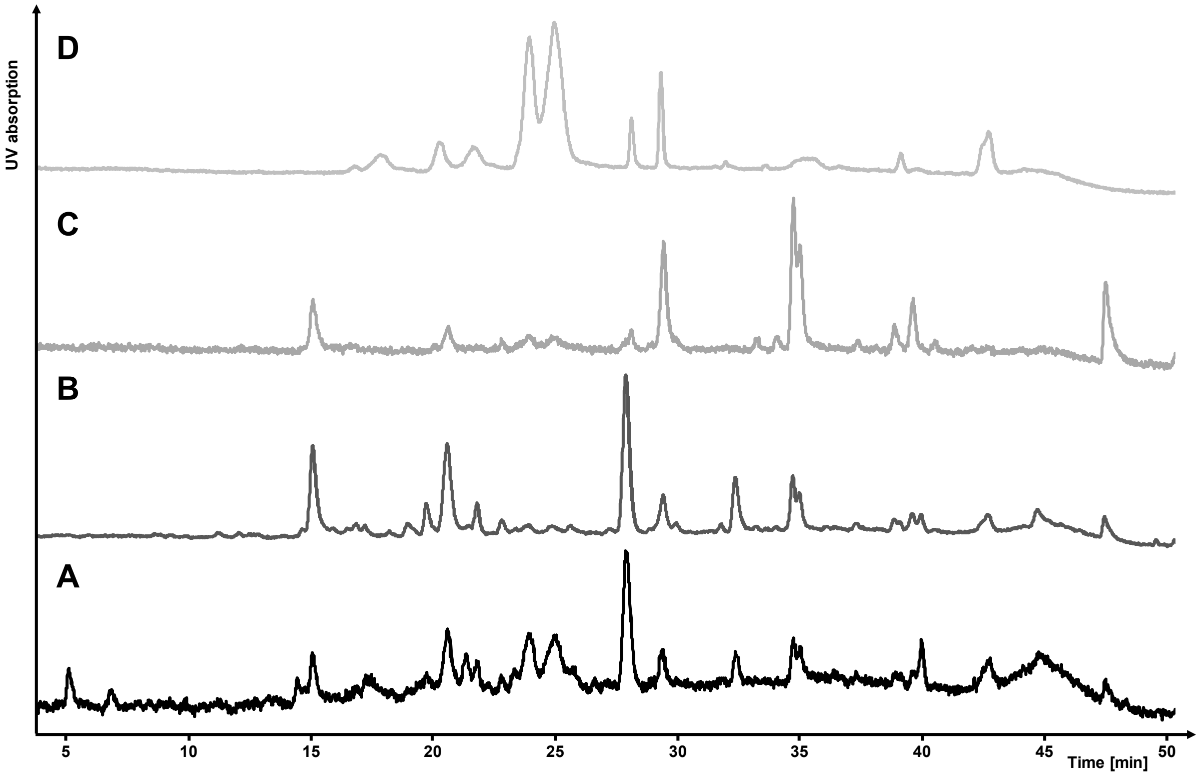

Figure S9. HPLC-UV chromatograms of the XAD-7 extract of red grape (Spain) at a wavelength of λ 280 nm (A), λ 320 nm (B), λ 360 nm (C), and λ 520 nm (D). Peak identification is given in Tables S16 and S17.

Table S16. HPLC-ESI-MS/MS data of the XAD-7 extract of red grape (Spain), identified anthocyanidins and their absorption maxima λ_{max}.

Peak No.	Retention time (min)	$[M]^+$ *m/z*	Fragments *m/z*	λ_{max} (nm)	Anthocyanin	Reference
A1	17.8	465	303	523/280	Delphinidin-3-glucoside	[28,31]
A2	20.4	449	287	516/280	Cyanidin-3-glucoside	[28,31]
A3	22.0	479	317	526/280	Petunidin-3-glucoside	[28,31]
A4	24.5	463	301	523/280	Peonidin-3-glucoside	[28,31]
A5	25.6	493	331	526/280	Malvidin-3-glucoside	[28,31]
A6	31.2	491	287	521/280	Cyanidin-3-(6′′-acetoyl)-glucoside	[31]
A7	32.1	521	317	526/280	Petunidin-3-(6′′-acetoyl)-glucoside	[28,31]
A8	35.0	505	301	523/280	Peonidin-3-(6′′-acetoyl)-glucoside	[28,31]
A9	35.8	535	331	528/280	Malvidin-3-(6′′-acetoyl)-glucoside	[28,31]
A10	36.8	611	303	523/280	Delphinidin-3-(6′′-coumaroyl)-glucoside	[31]
A11	37.7	801	639/331	525	Malvidin-3-(6′′-coumaroyl)-5-diglucoside	[31]
A12	39.6	595	287	521/280	Cyanidin-3-(6′′-coumaroyl)-glucoside	[28,31]
A13	40.1	625	479/317	524/280	Petunidin-3-(6′′-coumaroyl)-glucoside	[28,31]
A14	42.7	609	301	520/280	Peonidin-3-(6′′-coumaroyl)-glucoside	[28,31]
A15	43.0	639	331	530/280	Malvidin-3-(6′′-coumaroyl)-glucoside	[28,31]

Table S17. HPLC-ESI-MS/MS data of the XAD-7 extract of red grape (Spain), identified copigments and their absorption maxima λ_{max}.

Peak No.	Retention time (min)	$[M–H]^-$ *m/z*	Fragments *m/z*	λ_{max} (nm)	Copigment	Reference
1	5.2	169	125	278	Gallic acid	[28,31]
2	14.7	577	451/425/407/289	280	Procyanidin B_1	[28,31]
3	15.1	311	179/149	325	Caftaric acid	[28,31]
4	17.6	289	245/205/179	280	Catechin	[28,31]
5	19.7	325	265/235	314	Coumaric acid hexoside	[28,31]
6	20.5	295	163	312	Coutaric acid	[28,31]
7	21.0	577	451/425/407/289	280	Procyanidin B_2	[28,31]
8	22.7	289	245/205/179	280	Epicatechin	[28,31]
9	27.0	577	451/425/407/289	280	Procyanidin dimer	[28,31]
10	29.4	479	317/316	352	Myricetin-3-hexoside	[28,31]
11	33.1	609	301	356	Quercetin-3-rutinoside	[28,31]
12	34.6	463	301	352	Quercetin-3-glucoside	[28,31]
13	35.0	477	301	352	Quercetin-3-glucoronide	[28,31]
14	38.8	447	285/255	345	Kaempferol-3-glucoside	[28,31]

Sour cherry

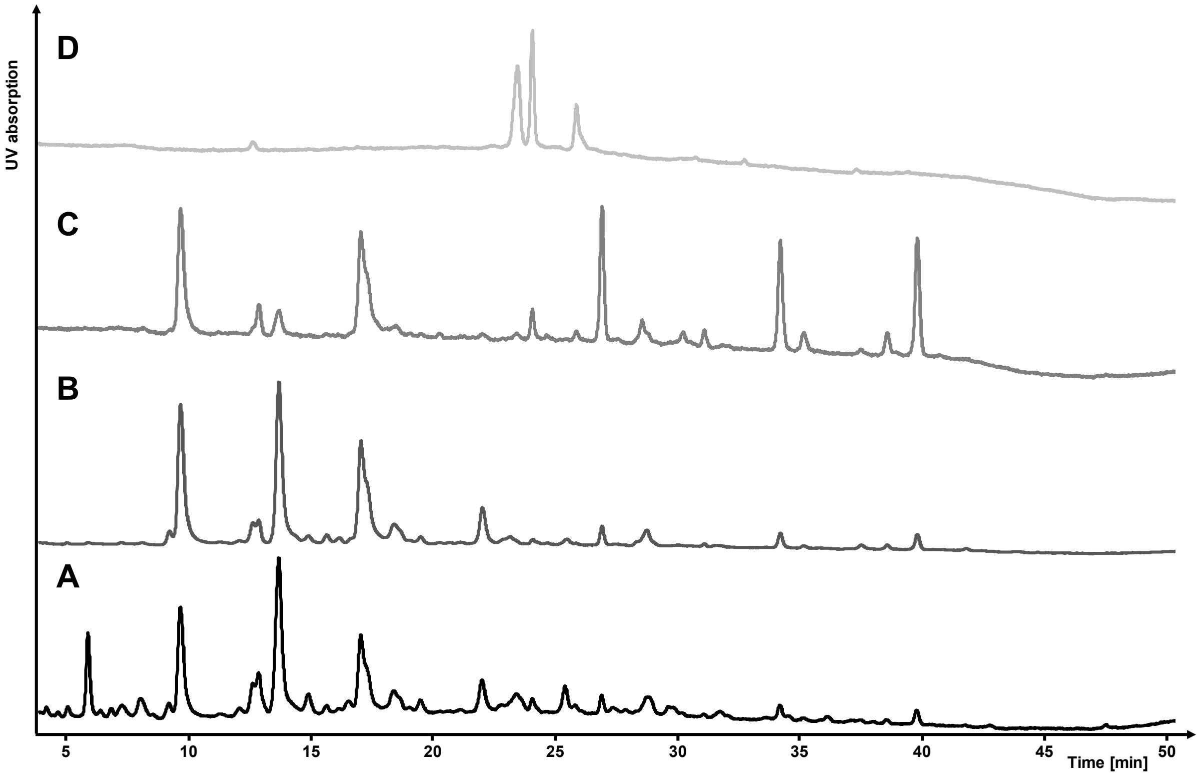

Figure S10. HPLC-UV chromatograms of the XAD-7 extract of sour cherry at a wavelength of λ 280 nm (A), λ 320 nm (B), λ 360 nm (C), and λ 520 nm (D). Peak identification is given in Tables S18 and S19.

Table S18. HPLC-ESI-MS/MS data of the XAD-7 extract of sour cherry, identified anthocyanidins and their absorption maxima λ_{max}.

Peak No.	Retention time (min)	$[M]^+$ *m/z*	Fragments *m/z*	λ_{max} (nm)	Anthocyanin	Reference
A1	22.5	611	287	519/280	Cyanidin-3-sophoroside	[28,31]
A2	23.5	757	611/287	520/280	Cyanidin-3-(2^G-glucosyl-rutinoside)	[28,31]
A3	26.0	595	449/287	517/280	Cyanidin-3-rutinoside	[28,31]

Table S19. HPLC-ESI-MS/MS data of the XAD-7 extract of sour cherry, identified copigments and their absorption maxima λ_{max}.

Peak No.	Retention time (min)	$[M–H]^-$ *m/z*	Fragments *m/z*	λ_{max} (nm)	Copigment	Reference
1	9.7	353	191/179/135	324	Neochlorogenic acid	[28,31]
2	13.7	337	163	312	Coumaroylquinic acid	[28,31]
3	17.1	353	191/179/161	325	Chlorogenic acid	[28,31]
4	21.9	289	245/205/179	280	Epicatechin	[28,31]
5	26.9	771	609/301	351	Quercetin-3-(2^G-glucosylrutinoside)	[28,31]
6	34.1	609	301	356	Quercetin-3-rutinoside	[28,31]
7	38.6	593	285/255	345	Kaempferol-3-rutinosid	[28,31]
8	39.8	623	315	354	Isorhamnetin-3-rutinosid	[28,31]

Strawberry

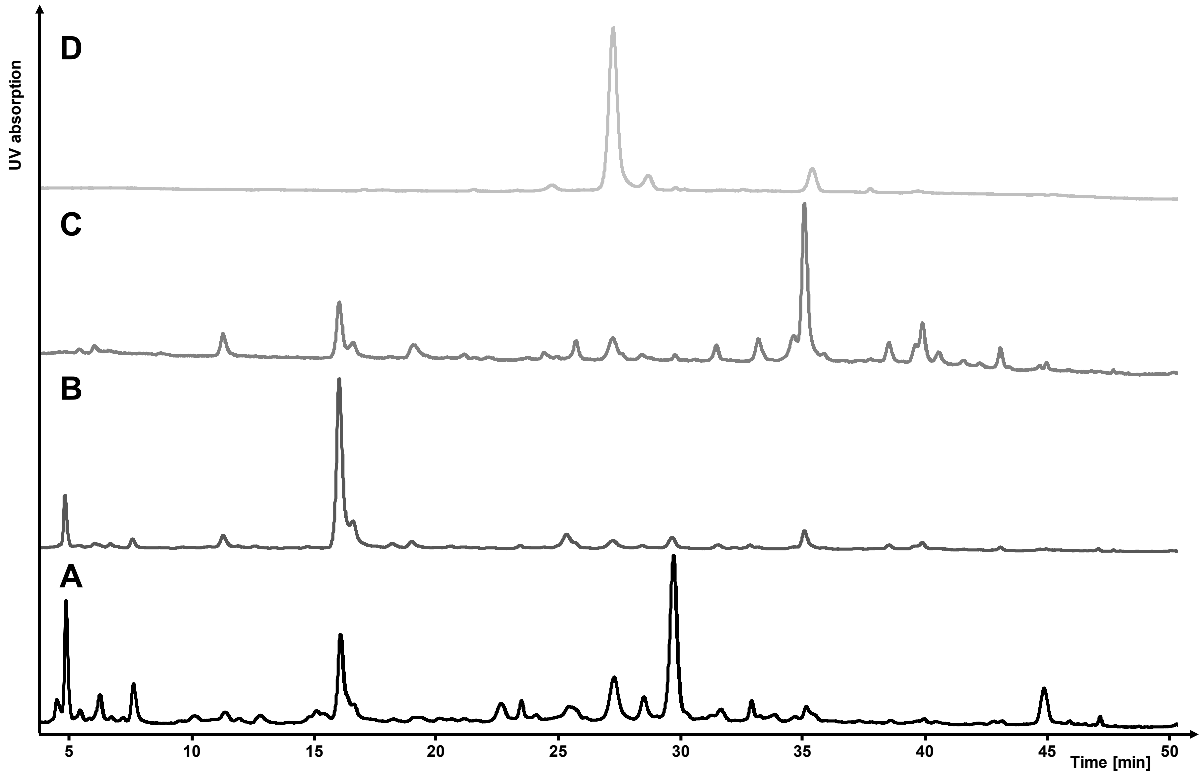

Figure S11. HPLC-UV chromatograms of the XAD-7 extract of strawberry at a wavelength of λ 280 nm (A), λ 320 nm (B), λ 360 nm (C), and λ 520 nm (D). Peak identification is given in Tables S20 and S21.

Table S20. HPLC-ESI-MS/MS data of the XAD-7 extract of strawberry, identified anthocyanidins and their absorption maxima λ_{max}.

Peak No.	Retention time (min)	$[M]^+$ *m/z*	Fragments *m/z*	λ_{max} (nm)	Anthocyanin	Reference
A1	24.8	449	287	516/280	Cyanidin-3-glucoside	[35]
A2	27.2	433	271	504/280	Pelargonidin-3-glucoside	[35]
A3	28.7	579	271	504/280	Pelargonidin-3-rutinoside	[35]
A4	35.4	519	271	506/280	Pelargonidin-3-(6′′-malonyl)-glucoside	[35]
A5	39.7	475	271	506/280	Pelargonidin-3-(6′′-acetoyl)-glucoside	[35]

Table S21. HPLC-ESI-MS/MS data of the XAD-7 extract of strawberry, identified copigments and their absorption maxima λ_{max}.

Peak No.	Retention time (min)	[M–H]⁻ *m*/*z*	Fragments *m*/*z*	λ_{max} (nm)	Copigment	Reference
1	6.2	783	481/301/257/229	350	Ellagitannin	[42]
2	7.2	299	239/179/137	262	Hydroxybenzoyl-hexsoside	[42]
3	10.2	783	481/301/257/229	350	Ellagitannin	[42]
4	11.4	341	179/161	313	Caffeolyhexose	[42]
5	12.8	577	451/425/407/289	280	Procyanidin B1	[42]
6	15.5	289	245/205/179	280	Catechin	[42]
7	16.1	325	163/145	320	*p*-Coumaroylhexose	[42]
8	25.7	449	355/287/193	312	Feruloylhexose	[42]
9	35.2	477	301	352	Quercetin-3-glucoronide	[42]
10	40.0	461	285	345	Kaempferol-3-glucuronide	[42]

Section S2: Cell Viability Testing

Resazurin reduction assay [54] was used to assess the relative viability of THP-1 cells after incubation with fruit beverage extracts. THP-1 cells were seeded in serum-reduced medium (composed as described in *2.1 Chemicals*) at a density of 200,000 (Figure S12) or 50,000 (Figure S13) THP-1 cells per well in a 96-well suspension plate (Sarstedt, Germany). Five hours after seeding, the cells were incubated for one hour with 50 µg/mL or 100 µg/mL DMSO-dissolved (final DMSO concentration per well: 0.1% DMSO) XAD-7 fruit extracts (done for each extract listed in *2.2 Samples*). 0.1% of DMSO without the addition of any XAD-7 fruit extract was used as negative control. Subsequently, the cells were co-stimulated overnight (18 hours) with 1 µg/mL lipopolysaccharide (LPS) and 10 ng/mL interferon-γ (IFN-γ). After their incubation at 37 °C, 5% CO_2, and 95% humidity the resazurin solution was added to reach a concentration of 44 µM resazurin (resazurin sodium salt diluted in sodium chloride phosphate buffer; Sigma Aldrich, Germany) per well. After 1 h of incubation at 37 °C, 5% CO_2 and 95% humidity, the resazurin-resorufin-conversion was analyzed fluorometrically, using excitation and emission wavelengths of 544 and 590 nm (Synergy H1, Agilent, USA). As relative cell viability is not reduced to 80% or less, no cytotoxicity due to the cell treatment is observed.

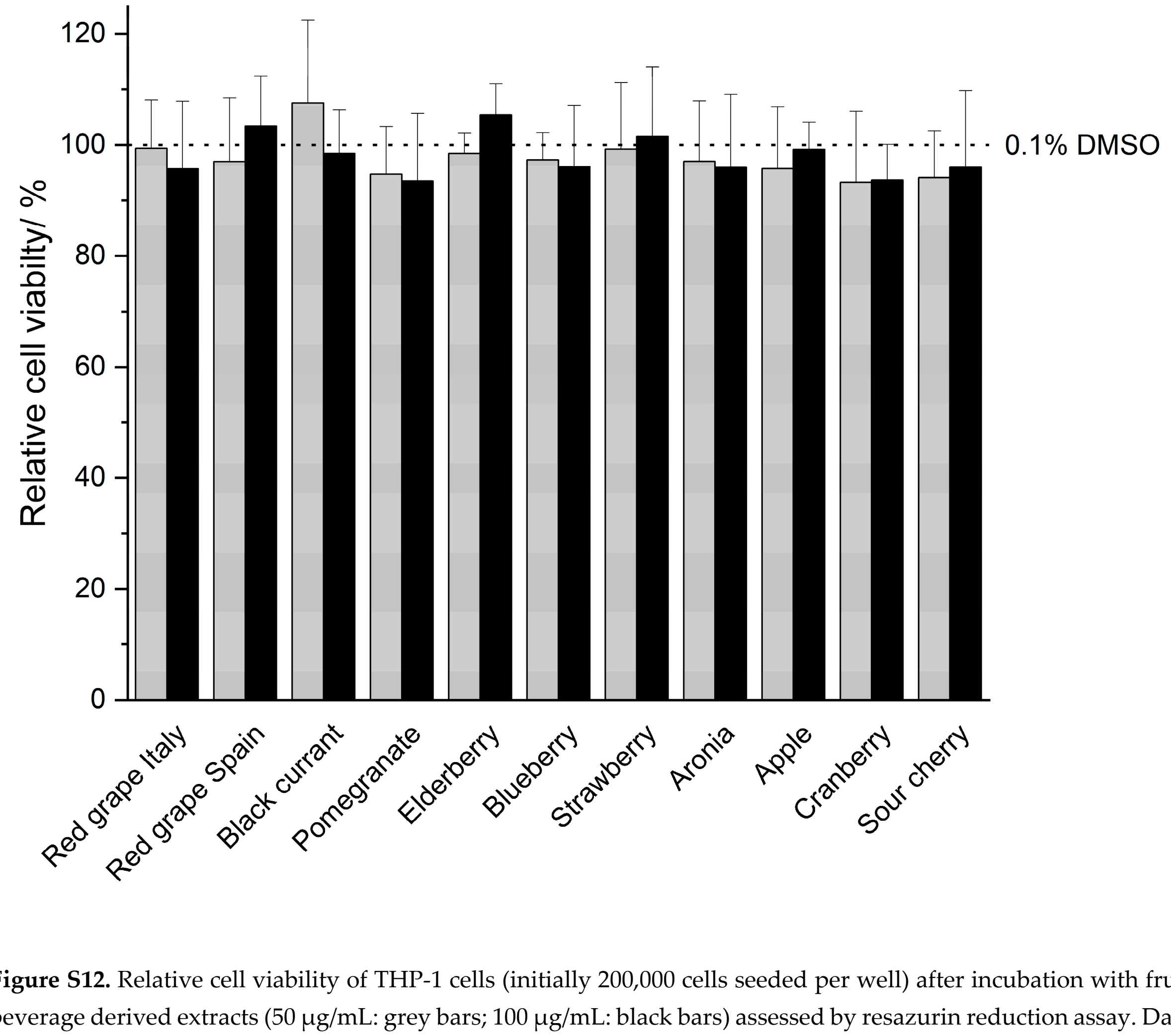

Figure S12. Relative cell viability of THP-1 cells (initially 200,000 cells seeded per well) after incubation with fruit beverage derived extracts (50 µg/mL: grey bars; 100 µg/mL: black bars) assessed by resazurin reduction assay. Data are shown as mean + SD relative to the 0.1% DMSO control, based on *n*=3 independent experiments.

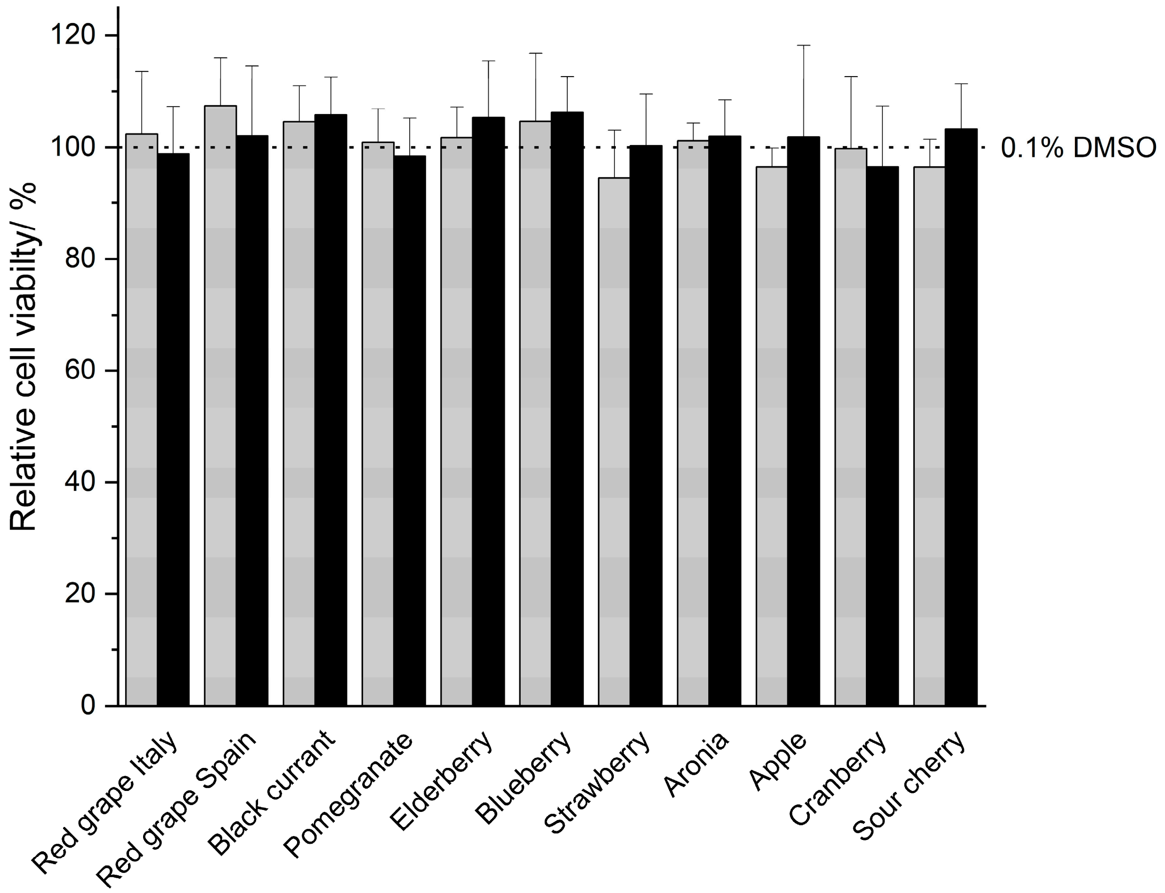

Figure S13. Relative cell viability of THP-1 cells (initially 50,000 cells seeded per well) after incubation with fruit beverage derived extracts (50 µg/mL: grey bars; 100 µg/mL: black bars) assessed by resazurin reduction assay. Data are shown as mean + SD relative to the 0.1% DMSO control, based on *n*=3 independent experiments.

Section S3: Statistical analysis

Table S22. Cytokine secretion after incubation of THP-1 cells with 50 µg/mL fruit beverage derived extracts assessed by Lumit® Immunoassay based on *n*=3 independent experiments (Figures 2–5). Statistical significance of the samples relative to the solvent control (0.1% DMSO) was determined using a two-sided Student's t-test, *p < 0.05.

XAD-7 extract	TNF-α	IL-1β	IL-6	IL-8
Red grape Italy	0.03522	0.01558	0.00028	0.00297
Red grape Spain	0.00378	0.02228	0.00145	0.00199
Black currant	0.00872	0.00436	0.01899	0.00322
Pomegranate	0.00122	0.01319	0.01174	0.00247
Elderberry	0.00821	0.02761	0.00328	0.00386
Blueberry	0.01773	0.02819	0.01295	0.01275
Strawberry	0.00104	0.01993	0.00552	0.03911
Aronia	0.02321	0.04554	0.14157	0.03008
Apple	0.00014	0.00804	0.04593	0.05585
Cranberry	0.00567	0.01258	0.00318	0.03216
Sour cherry	0.01652	0.04544	0.00193	0.01033

Table S23. Cytokine secretion after incubation of THP-1 cells with 100 µg/mL fruit beverage derived extracts assessed by Lumit® Immunoassay based on *n*=3 independent experiments (Figures 2–5). Statistical significance of the samples relative to the solvent control (0.1% DMSO) was determined using a two-sided Student's t-test. *p < 0.05

XAD-7 extract	TNF-α	IL-1β	IL-6	IL-8
Red grape Italy	0.01375	0.00311	0.00001	0.00005
Red grape Spain	0.00399	0.03991	0.00014	0.00050
Black currant	0.01446	0.00031	0.00652	0.00370
Pomegranate	0.02798	0.00482	0.00354	0.00242
Elderberry	0.01256	0.00655	0.00069	0.00953
Blueberry	0.00163	0.00637	0.00261	0.00077
Strawberry	0.00844	0.01769	0.00727	0.00689
Aronia	0.00134	0.38351	0.39938	0.00959
Apple	0.01625	0.00875	0.25067	0.15844
Cranberry	0.00989	0.00810	0.00382	0.00706
Sour cherry	0.00715	0.01683	0.00420	0.03589

Section S4: Cross-extract comparison regarding their anti-inflammatory effects

Table S24. Mean and SD of cytokine secretion after incubation of THP-1 cells with 50 μg/mL fruit beverage derived extracts assessed by Lumit® Immunoassay based on *n*=3 independent experiments (Figures 2–5).

XAD-7 extract	TNF-α mean	TNF-α SD	IL-1β mean	IL-1β SD	IL-6 mean	IL-6 SD	IL-8 mean	IL-8 SD
Red grape Italy	39.20	20.31	51.98	10.50	21.83	3.14	28.09	6.80
Red grape Spain	34.31	7.01	53.12	12.33	34.72	3.82	44.40	4.30
Black currant	42.99	9.28	38.50	7.06	49.10	11.50	59.83	3.96
Pomegranate	46.54	3.24	50.14	10.02	50.38	13.26	60.35	3.42
Elderberry	47.74	8.25	55.26	13.15	49.52	5.56	65.16	3.76
Blueberry	49.87	11.72	53.91	13.70	51.56	13.07	68.12	6.30
Strawberry	49.65	2.82	57.14	10.64	44.81	3.58	69.31	10.83
Aronia	46.56	14.35	79.33	7.91	58.11	18.35	87.29	3.91
Apple	44.85	1.14	61.72	5.98	58.48	8.55	84.40	6.67
Cranberry	52.12	6.27	60.55	7.74	58.21	1.50	76.92	7.35
Sour cherry	47.07	11.93	63.20	14.07	48.65	6.51	74.39	4.54

Table S25. Mean and SD of cytokine secretion after incubation of THP-1 cells with 100 μg/mL fruit beverage derived extracts assessed by Lumit® Immunoassay based on *n*=3 independent experiments (Figures 2–5).

XAD-7 extract	TNF-α mean	TNF-α SD	IL-1β mean	IL-1β SD	IL-6 mean	IL-6 SD	IL-8 mean	IL-8 SD
Red grape Italy	20.27	16.36	33.75	6.42	11.46	0.76	11.84	1.04
Red grape Spain	21.21	8.64	53.15	16.71	17.50	3.10	20.67	3.07
Black currant	32.39	14.24	14.46	2.61	29.98	19.42	34.32	6.94
Pomegranate	43.66	16.68	27.45	8.76	35.15	12.69	41.25	5.02
Elderberry	39.60	11.84	39.19	8.57	37.93	8.49	59.39	6.92
Blueberry	31.27	4.81	43.22	7.88	36.61	10.03	49.92	2.41
Strawberry	40.66	9.50	52.92	10.99	36.37	1.73	54.54	6.57
Aronia	49.97	3.18	114.85	23.24	66.02	37.03	48.06	8.87
Apple	41.04	13.18	63.47	5.96	71.56	34.37	91.21	6.91
Cranberry	45.25	9.50	58.89	6.45	53.89	8.17	67.37	4.77
Sour cherry	49.70	7.41	52.96	10.71	37.39	3.47	76.01	8.09

References

24. Niesen, S.; Göttel, C.; Becker, H.; Bakuradze, T.; Winterhalter, P.; Richling, E. Fractionation of Extracts from Black Chokeberry, Cranberry, and Pomegranate to Identify Compounds That Influence Lipid Metabolism. *Foods* **2022,** *11* (4). DOI: 10.3390/foods11040570.
28. Köpsel, M.; Kostka, T.; Niesen, S.; Winterhalter, P.; Esatbeyoglu, T. Influence of fractionation of polyphenols by membrane chromatography on antioxidant, antimicrobial and proliferation-inhibiting effects of red fruit juices. *Food Chemistry* **2025,** *463* (Pt 2), 141216. DOI: 10.1016/j.foodchem.2024.141216.
29. Gerasimov, M. A.; Perova, I. B.; Eller, K. I.; Akimov, M. Y.; Sukhanova, A. M.; Rodionova, G. M.; Ramenskaya, G. V. Investigation of Polyphenolic Compounds in Different Varieties of Black Chokeberry Aronia melanocarpa. *Molecules* **2023,** *28* (10). DOI: 10.3390/molecules28104101.
30. Oszmiański, J.; Lachowicz, S. Effect of the Production of Dried Fruits and Juice from Chokeberry (Aronia melanocarpa L.) on the Content and Antioxidative Activity of Bioactive Compounds. *Molecules* **2016,** *21* (8). DOI: 10.3390/molecules21081098.
31. Göttel, C.; Niesen, S.; Daub, V.; Werle, T.; Bakuradze, T.; Winterhalter, P.; Richling, E. In Vitro Inhibition of Phosphodiesterase 3B (PDE 3B) by Anthocyanin-Rich Fruit Juice Extracts and Selected Anthocyanins. *International journal of molecular sciences* **2020,** *21* (18). DOI: 10.3390/ijms21186934.
32. Gavrilova, V.; Kajdzanoska, M.; Gjamovski, V.; Stefova, M. Separation, characterization and quantification of phenolic compounds in blueberries and red and black currants by HPLC-DAD-ESI-MSn. *Journal of agricultural and food chemistry* **2011,** *59* (8), 4009–4018. DOI: 10.1021/jf104565y.
33. Wang, Y.; Fong, S. K.; Singh, A. P.; Vorsa, N.; Johnson-Cicalese, J. Variation of Anthocyanins, Proanthocyanidins, Flavonols, and Organic Acids in Cultivated and Wild Diploid Blueberry Species. *horts* **2019,** *54* (3), 576–585. DOI: 10.21273/HORTSCI13491-18.
34. Mena, P.; Calani, L.; Dall'Asta, C.; Galaverna, G.; García-Viguera, C.; Bruni, R.; Crozier, A.; Del Rio, D. Rapid and comprehensive evaluation of (poly)phenolic compounds in pomegranate (Punica granatum L.) juice by UHPLC-MSn. *Molecules* **2012,** *17* (12), 14821–14840. DOI: 10.3390/molecules171214821.
35. Da Silva, F. L.; Escribano-Bailón, M. T.; Pérez Alonso, J. J.; Rivas-Gonzalo, J. C.; Santos-Buelga, C. Anthocyanin pigments in strawberry. *LWT - Food Science and Technology* **2007,** *40* (2), 374–382. DOI: 10.1016/j.lwt.2005.09.018.
36. Kahle, K.; Kraus, M.; Richling, E. Polyphenol profiles of apple juices. *Molecular nutrition & food research* **2005,** *49* (8), 797–806. DOI: 10.1002/mnfr.200500064.
37. Verdu, C. F.; Gatto, J.; Freuze, I.; Richomme, P.; Laurens, F.; Guilet, D. Comparison of two methods, UHPLC-UV and UHPLC-MS/MS, for the quantification of polyphenols in cider apple juices. *Molecules* **2013,** *18* (9), 10213–10227. DOI: 10.3390/molecules180910213.
38. Schuster, B.; Herrmann, K. Hydroxybenzoic and hydroxycinnamic acid derivatives in soft fruits. *Phytochemistry* **1985,** *24* (11), 2761–2764. DOI: 10.1016/S0031-9422(00)80722-0.
39. Määttä, K. R.; Kamal-Eldin, A.; Törrönen, A. R. High-performance liquid chromatography (HPLC) analysis of phenolic compounds in berries with diode array and electrospray ionization mass spectrometric (MS) detection: ribes species. *Journal of agricultural and food chemistry* **2003,** *51* (23), 6736–6744. DOI: 10.1021/jf0347517.
40. Grace, M. H.; Esposito, D.; Dunlap, K. L.; Lila, M. A. Comparative analysis of phenolic content and profile, antioxidant capacity, and anti-inflammatory bioactivity in wild Alaskan and commercial Vaccinium berries. *Journal of agricultural and food chemistry* **2014,** *62* (18), 4007–4017. DOI: 10.1021/jf403810y.
41. Uzlasir, T.; Kadiroglu, P.; Selli, S.; Kelebek, H. LC-DAD-ESI-MS/MS characterization of elderberry flower (Sambucus nigra) phenolic compounds in ethanol, methanol, and aqueous extracts. *J Food Process Preserv* **2021,** *45* (8). DOI: 10.1111/jfpp.14478.
42. Kajdžanoska, M.; Gjamovski, V.; Stefova, M. HPLC-DAD-ESI-MSn identification of phenolic compounds in cultivated strawberries from Macedonia. *Maced. J. Chem. Chem. Eng.* **2013,** *29* (2), 181. DOI: 10.20450/mjcce.2010.165.
54. O'Brien, J.; Wilson, I.; Orton, T.; Pognan, F. Investigation of the Alamar Blue (Resazurin) Fluorescent Dye for the Assessment of Mammalian Cell Cytotoxicity. *Eur. J. Biochem.* **2000**, *267* (17), 5421–5426. https://doi.org/10.1046/j.1432-1327.2000.01606.x.
